# Malaria Surveillance — United States, 2017

**DOI:** 10.15585/mmwr.ss7002a1

**Published:** 2021-03-19

**Authors:** Kimberly E. Mace, Naomi W. Lucchi, Kathrine R. Tan

**Affiliations:** 1 Malaria Branch, Division of Parasitic Diseases and Malaria, Center for Global Health, CDC

## Abstract

**Problem/Condition:**

Malaria in humans is caused by intraerythrocytic protozoa of the genus *Plasmodium*. These parasites are transmitted by the bite of an infective female *Anopheles* species mosquito. The majority of malaria infections in the United States occur among persons who have traveled to regions with ongoing malaria transmission. However, malaria is occasionally acquired by persons who have not traveled out of the country through exposure to infected blood products, congenital transmission, nosocomial exposure, or local mosquitoborne transmission. Malaria surveillance in the United States is conducted to provide information on its occurrence (e.g., temporal, geographic, and demographic), guide prevention and treatment recommendations for travelers and patients, and facilitate rapid transmission control measures if locally acquired cases are identified.

**Period Covered:**

This report summarizes confirmed malaria cases in persons with onset of illness in 2017 and trends in previous years.

**Description of System:**

Malaria cases diagnosed by blood film microscopy, polymerase chain reaction, or rapid diagnostic tests are reported to local and state health departments through electronic laboratory reports or by health care providers or laboratory staff members. Case investigations are conducted by local and state health departments, and reports are transmitted to CDC through the National Malaria Surveillance System (NMSS), the National Notifiable Diseases Surveillance System (NNDSS), or direct CDC consultations. CDC reference laboratories provide diagnostic assistance and conduct antimalarial drug resistance marker testing on blood samples submitted by health care providers or local or state health departments. This report summarizes data from the integration of all cases from NMSS and NNDSS, CDC reference laboratory reports, and CDC clinical consultations.

**Results:**

CDC received reports of 2,161 confirmed malaria cases with onset of symptoms in 2017, including two congenital cases, three cryptic cases, and two cases acquired through blood transfusion. The number of malaria cases diagnosed in the United States has been increasing since the mid-1970s; in 2017, the number of cases reported was the highest in 45 years, surpassing the previous peak of 2,078 confirmed cases reported in 2016. Of the cases in 2017, a total of 1,819 (86.1%) were imported cases that originated from Africa; 1,216 (66.9%) of these came from West Africa. The overall proportion of imported cases originating from West Africa was greater in 2017 (57.6%) than in 2016 (51.6%). Among all cases, *P. falciparum* accounted for the majority of infections (1,523 [70.5%]), followed by *P. vivax* (216 [10.0%]), *P. ovale* (119 [5.5%]), and *P. malariae* (55 [2.6%]). Infections by two or more species accounted for 22 cases (1.0%). The infecting species was not reported or was undetermined in 226 cases (10.5%). CDC provided diagnostic assistance for 9.5% of confirmed cases and tested 8.0% of specimens with *P. falciparum* infections for antimalarial resistance markers. Most patients (94.8%) had symptom onset <90 days after returning to the United States from a country with malaria transmission. Of the U.S. civilian patients who reported reason for travel, 73.1% were visiting friends and relatives. The proportion of U.S. residents with malaria who reported taking any chemoprophylaxis in 2017 (28.4%) was similar to that in 2016 (26.4%), and adherence was poor among those who took chemoprophylaxis. Among the 996 U.S. residents with malaria for whom information on chemoprophylaxis use and travel region were known, 93.3% did not adhere to or did not take a CDC-recommended chemoprophylaxis regimen. Among 805 women with malaria, 27 reported being pregnant. Of these, 10 pregnant women were U.S. residents, and none reported taking chemoprophylaxis to prevent malaria. A total of 26 (1.2%) malaria cases occurred among U.S. military personnel in 2017, fewer than in 2016 (41 [2.0%]). Among all reported cases in 2017, a total of 312 (14.4%) were classified as severe malaria illnesses, and seven persons died. In 2017, CDC analyzed 117 *P. falciparum*-positive and six *P. falciparum* mixed-species samples for antimalarial resistance markers (although certain loci were untestable in some samples); identification of genetic polymorphisms associated with resistance to pyrimethamine were found in 108 (97.3%), to sulfadoxine in 77 (69.4%), to chloroquine in 38 (33.3%), to mefloquine in three (2.7%), and to atovaquone in three (2.7%); no specimens tested contained a marker for artemisinin resistance. The data completeness of key variables (species, country of acquisition, and resident status) was lower in 2017 (74.4%) than in 2016 (79.4%).

**Interpretation:**

The number of reported malaria cases in 2017 continued a decades-long increasing trend, and for the second year in a row the highest number of cases since 1971 have been reported. Despite progress in malaria control in recent years, the disease remains endemic in many areas globally. The importation of malaria reflects the overall increase in global travel to and from these areas. Fifty-six percent of all cases were among persons who had traveled from West Africa, and among U.S. civilians, visiting friends and relatives was the most common reason for travel (73.1%). Frequent international travel combined with the inadequate use of prevention measures by travelers resulted in the highest number of imported malaria cases detected in the United States in 4 decades.

**Public Health Actions:**

The best way to prevent malaria is to take chemoprophylaxis medication during travel to a country where malaria is endemic. Adherence to recommended malaria prevention strategies among U.S. travelers would reduce the numbers of imported cases; reasons for nonadherence include prematurely stopping after leaving the area where malaria was endemic, forgetting to take the medication, and experiencing a side effect. Travelers might not understand the risk that malaria poses to them; thus, health care providers should incorporate risk education to motivate travelers to be adherent to chemoprophylaxis. Malaria infections can be fatal if not diagnosed and treated promptly with antimalarial medications appropriate for the patient’s age, medical history, the likely country of malaria acquisition, and previous use of antimalarial chemoprophylaxis. Antimalarial use for chemoprophylaxis and treatment should be informed by the most recent guidelines, which are frequently updated. In 2018, two formulations of tafenoquine (i.e., Arakoda and Krintafel) were approved by the Food and Drug Administration (FDA) for use in the United States. Arakoda was approved for use by adults for chemoprophylaxis; the regimen requires a predeparture loading dose, taking the medication weekly during travel, and a short course posttravel. The Arakoda chemoprophylaxis regimen is shorter than alternative regimens, which could possibly improve adherence. This medication also might prevent relapses. Krintafel was approved for radical cure of *P. vivax* infections in those aged >16 years and should be co-administered with chloroquine (https://www.cdc.gov/malaria/new_info/2020/tafenoquine_2020.html). In April 2019, intravenous artesunate became the first-line medication for treatment of severe malaria in the United States. Artesunate was recently FDA approved but is not yet commercially available. The drug can be obtained from CDC under an investigational new drug protocol. Detailed recommendations for preventing malaria are available to the general public at the CDC website (https://www.cdc.gov/malaria/travelers/drugs.html). Health care providers should consult the CDC *Guidelines for Treatment of Malaria in the United States* and contact the CDC’s Malaria Hotline for case management advice when needed. Malaria treatment recommendations are available online (https://www.cdc.gov/malaria/diagnosis_treatment) and from the Malaria Hotline (770-488-7788 or toll-free 855-856-4713). Persons submitting malaria case reports (care providers, laboratories, and state and local public health officials) should provide complete information because incomplete reporting compromises case investigations and efforts to prevent infections and examine trends in malaria cases. Molecular surveillance of antimalarial drug resistance markers (https://www.cdc.gov/malaria/features/ars.html) enables CDC to track, guide treatment, and manage drug resistance in malaria parasites both domestically and internationally. More samples are needed to improve the completeness of antimalarial drug resistance analysis; therefore, CDC requests that blood specimens be submitted for any case of malaria diagnosed in the United States.

## Introduction

Malaria parasites of the *Plasmodium* genus are transmitted through the bite of infective mosquitoes. Female *Anopheles* species mosquitoes transmit four *Plasmodium* species that commonly cause illness in humans: *P. falciparum*, *P. vivax, P. ovale*, and *P. malariae.* Mixed infections with multiple species are possible and occur in areas where more than one species is in circulation (*1*). Rarely, humans can be infected with *P. knowlesi,* a predominantly simian malaria found in Southeast Asia.

In 2017, malaria was endemic in 90 countries and territories in the tropics and subtropics, with approximately half the world population at risk for infection. The World Health Organization (WHO) estimated that 219 million cases of malaria occurred worldwide in 2017, resulting in an estimated 400,000 deaths (*2*). Since 2000, the global community has funded and implemented malaria control efforts and achieved an estimated 20 million fewer malaria cases in 2017 alone, compared with 2010, and cumulatively, these efforts have prevented millions of malaria deaths. However, during 2015–2017, the number of cases globally was stable (*2*). *P. falciparum* and *P. vivax* contribute the most morbidity worldwide. *P. falciparum* has the highest prevalence in sub-Saharan Africa and is the parasite most commonly associated with severe illness and death, typically among children aged <5 years. The African region accounts for an estimated 92% of malaria cases worldwide; 99.7% of cases in Africa are caused by *P. falciparum,* and 93% of all malaria deaths occur in Africa (*2*). *P. vivax* accounted for 3.4% of estimated malaria cases in 2017 and has a broader geographic range than *P. falciparum. P. vivax* accounts for an estimated 74% of malaria infections in the Americas, 37% in Southeast Asia, and 31% in the Eastern Mediterranean region (*2*). In some temperate regions, the interval between inoculation and the onset of first symptoms of *P. vivax* infection can be 6 months or more (*3*–*5*). Malaria relapses are common with *P. vivax* and *P. ovale* parasites, which have dormant liver stages (hypnozoites) that can reactivate months or years after the acute infection. *P. malariae* parasites are found throughout the tropics and subtropics, account for a minority of the *Plasmodium* parasites in circulation, and are often detected in mixed-species infections. *P. malariae* parasites mature slowly in human and mosquito hosts and, although they do not typically cause severe symptoms in humans, can result in persistent low-density infections that can last for years or even a lifetime, providing opportunities for ongoing transmission and health sequelae (*6*,*7*).

Malaria was eliminated from the United States* in 1951 (*8*), although the *Anopheles* mosquito vector exists in many states (*9*). Since 1957, malaria surveillance has been supported to detect cases and prevent reintroduction, monitor antimalarial resistance, assess trends in case acquisition, and guide malaria prevention and treatment recommendations for U.S. residents. Most malaria cases diagnosed in the United States are imported from countries with ongoing mosquitoborne transmission. Occasionally, congenitally acquired cases, induced cases resulting from exposure to blood products, and nonimported cases for which exposure cannot be easily explained occur. State and local health departments and CDC investigate cryptic cases. During 2000–2017, a total of 11 instances of transfusion-transmitted malaria have been identified in the United States (*10*–*17*). Since 2010, one case each occurred in 2011 and 2016 (*11*,*13*); two cases occurring in 2017 are described in this report. During 1957–2003, a total of 63 malaria outbreaks occurred in the United States. The last well-documented local mosquitoborne transmission occurred in 2003, when eight cases were diagnosed among nontravelers in Palm Beach, Florida (*18*–*20*).

Clinical illness results from the presence of an asexual, intraerythrocytic stage of the parasite in red blood cells, and symptom severity ranges from absent or mild symptoms to severe illness and death. Factors that contribute to variability in illness severity are complex and include the parasite species, the patient’s age and immune response to the infection, the presence of acquired or protective immunity, the patient’s general health and nutritional constitution, chemoprophylaxis effects, and time to initiate appropriate treatment (*6*). Persons that live in areas with high malaria transmission, who experience repeated malarial illnesses, might develop partial protective immunity that can result in less severe illness or even asymptomatic parasitemia. However, without continual exposure, this semi-immunity will be lost within a few years (*21**–**23*); thus, it is assumed that U.S. residents do not have any degree of protective immunity to malaria and are susceptible to severe illness. Although malaria symptoms vary by age and immunologic status, the majority of patients have fever (*24*). Symptoms associated with uncomplicated malaria include chills, sweating, headache, fatigue, myalgia, cough, nausea, and mild anemia. If not treated promptly, malaria can rapidly progress and affect multiple organ systems and result in altered consciousness (cerebral malaria), seizures, severe anemia, acute kidney injury and liver failure, respiratory distress, coma, permanent disability, and death. Travel history should be routinely requested for patients with fever. Malaria should be considered in the differential diagnosis for all persons who have fever and who recently traveled to areas where malaria is endemic as well as for persons who have unexplained fever, regardless of travel history.

To prevent malaria, CDC recommends that U.S. residents use chemoprophylaxis: antimalarial medication taken before, during, and after travel to a country with malaria transmission. Persons who intend to travel should ask their physician for a prescription for an antimalarial that is appropriate for the region of travel, the age of the patient, pregnancy status, and individual preferences (e.g., cost or regimen type [daily or weekly]). CDC provides chemoprophylaxis guidelines to health care providers and the public, and links to these resources can be found at the end of this report. Although avoiding mosquito bites is not possible in many transmission settings, travelers should consider mosquito avoidance measures, specifically the use of repellents, treating clothing with permethrin, sleeping in screened sleep spaces, and using an insecticide-treated bed net (https://www.cdc.gov/malaria/travelers/index.html).

This report summarizes malaria cases reported to CDC with onset of symptoms in 2017, describes trends during previous years, and highlights information on risk factors and prevention. The intended audience includes public health authorities, health care providers, and persons traveling to areas with malaria transmission. Information on chemoprophylaxis, diagnosis, and treatment is provided for health care professionals and the public, and links to additional malaria information and resources are provided.

## Methods

### Data Sources and Analysis

Malaria case reports were submitted to CDC through the National Malaria Surveillance System (NMSS) and the National Notifiable Diseases Surveillance System (NNDSS) (*25*). The Armed Forces Health Surveillance Branch provides reports of malaria among military members to NMSS. As a notifiable condition, positive malaria laboratory tests are automatically reported from hospital, commercial, public health, and other laboratories to state and local health departments through the electronic laboratory reporting system (*26*). The electronic laboratory reports prompt investigations by state and local health departments that are submitted to CDC via NNDSS and NMSS. Both systems rely on passive reporting from the jurisdictions, and the number of cases might differ (e.g., because of differences in date classifications). NNDSS report dates are assigned according to the date reported to the health department, and NMSS assigns dates according to illness onset. In addition, NNDSS provides only basic case demographic information, whereas NMSS collects detailed epidemiologic data, including laboratory results, travel history, and clinical history, which facilitate investigation and classification of each case. Some cases also are reported through direct consultation with CDC staff via the Malaria Hotline. Diagnostic confirmation of cases often is facilitated by the CDC reference laboratory. This report summarizes data from the integration of all NMSS and NNDSS cases and CDC reference laboratory reports after deduplication and reconciliation.

Malaria cases are classified as confirmed or suspected using the 2014 case definition from the Council of State and Territorial Epidemiologists and CDC (*27*). Malaria cases are further categorized by infecting species. When more than a single species is detected, the case is categorized as a mixed infection. All categories are mutually exclusive. Diagnosis of malaria is made by blood film microscopy or polymerase chain reaction (PCR). A rapid diagnostic test (RDT) can be used to detect malaria antigens (*28*); however, the diagnosis must be confirmed either by microscopy or PCR to be counted as a case. Only data from confirmed cases are included in this report.

CDC staff members review all reports when received and request any additional information needed from the provider or the jurisdiction. Rare cases classified as acquired in the United States are investigated further, as are those classified as induced, congenital, or cryptic according to the definitions that follow. The malaria case report form and instructions for completing it are available from the CDC malaria website (*29*). Data from the structured malaria case report form were entered into the NMSS Access database by a subject matter expert dedicated to malaria surveillance; alternatively, spreadsheets extracted from department of health surveillance systems were normalized by the malaria surveillance subject matter expert and imported into NMSS. Data elements analyzed include age, sex, pregnancy status, residence, illness onset date, laboratory results (test type, species, and parasitemia percentage), travel history (countries, regions, and dates), chemoprophylaxis (medication used and adherence), history of malaria (date and species), blood transfusion or organ transplant history, clinical complications, treatment medications, illness outcome (survived versus died), and case classification. Data elements with missing values were excluded from analysis.

The chi-square test was used to calculate p values and assess differences between variables reported in 2017 compared with previous years. A p value of <0.05 was considered statistically significant. Linear regression using the least-squares method was used to assess the linear trend in the number of cases during 1973–2017. The Pearson product-moment correlation coefficient (R^2^) was used to describe the proportion of variation explained by the model. States with one or more cases were categorized into quartiles using the QNTLDEF=5 option in SAS (version 9.4; SAS Institute).

### Definitions

The following definitions are used in malaria surveillance for the United States (*27*,*29*):

- **Adherence to chemoprophylaxis:** Reported response (yes or no) to the question, “Was chemoprophylaxis taken as prescribed?”
- **Confirmed case:** Symptomatic or asymptomatic infection that occurs in a person in the United States or one of its territories who has laboratory-confirmed (by microscopy or PCR) malaria parasitemia, regardless of whether the person had previous episodes of malaria while in other countries. A subsequent episode of malaria is counted as an additional case, regardless of the detected *Plasmodium* species, unless the case is indicated as a treatment failure within 4 weeks of initial presentation.
- **Laboratory criteria for diagnosis:** Demonstration of malaria parasites by microscopy or PCR (including as confirmation of an initial positive RDT).
- **Non-U.S. residents:** Persons who are residents of a country other than the United States. Immigrants and refugees who are establishing residence in the United States are classified as non-U.S. residents if their exposure occurred while they were residents in their originating country.
- **Suspect case:** A positive malaria RDT result in a person in the United States or one of its territories without confirmation by microscopy or PCR, regardless of whether the person experienced previous episodes of malaria while in other countries.
- **Transfusion transmitted malaria (TTM):** A *Plasmodium* infection that is accidentally caused by the transfusion of whole blood or blood components from an infected donor to a recipient (*30*). In NMSS, the recipient is classified as having induced malaria.
- **Treatment according to CDC recommendations (i.e., appropriate treatment):** Treated with a CDC-recommended regimen appropriate for species, region, and severity of disease (*31*). Patients who received more antimalarial medication than recommended were classified as appropriately treated because the precise sequence and circumstances of excess treatment are not included in the malaria case report and characterizing the purpose or appropriateness of the additional antimalarial treatment is often not possible.
- **U.S. civilians:** Any U.S. residents, excluding U.S. military personnel.
- **U.S. residents:** Persons who live in the United States, including both civilian and U.S. military personnel, regardless of legal citizenship. This category does not include recent refugees or immigrants who are establishing residence in the United States if their exposure occurred when they were residents of their originating country.

This report also uses terms derived from WHO recommendations (*32*) and the CDC *Yellow Book* (*33*). Definitions of the following terms are included for reference:

- **Congenital malaria:** Malaria infection transmitted directly from mother to child during pregnancy or childbirth.
- **Cryptic malaria:** A case of malaria for which epidemiologic investigations cannot identify a plausible mode of acquisition.
- **Imported malaria:** Malaria acquired outside a specific area. In this report, imported cases are those acquired outside the United States and its territories.
- **Indigenous malaria:** Local mosquitoborne transmission of malaria with no evidence of importation and no direct link to transmission from an imported case.
- **Induced malaria:** Malaria transmission through a blood transfusion, organ transplantation, or another parenteral route, not mosquitoborne or congenital transmission.
- **Introduced malaria:** Local mosquitoborne transmission of malaria with strong epidemiological evidence linking the case to an imported case.
- **Radical treatment (or radical cure):** Treatment to kill dormant liver-stage parasites (hypnozoites) of *P. vivax* and *P. ovale* to prevent relapses of malaria.
- **Relapsing malaria:** Recurrence of disease after it has been apparently cured. In malaria, true relapses are caused by activation of dormant liver-stage parasites (hypnozoites) of *P. vivax* and *P. ovale* only. Hypnozoite activation is typically delayed after the primary exposure (*34*); therefore, likely relapses of *P. vivax* and *P. ovale* are defined as occurring >45 days after travel to an area where malaria is endemic.
- **Severe malaria:** A case of malaria with one or more of the following manifestations: neurologic symptoms, acute kidney injury, severe anemia (hemoglobin [Hb] <7g/dL), acute respiratory distress syndrome (ARDS), jaundice, or ≥5% parasitemia (*35*,*36*). Cases also were counted as severe if the person received treatment for severe malaria (i.e., artesunate, quinidine, or an exchange transfusion) despite having no specific severe manifestations reported. All fatal cases, for which malaria was the cause of death, were classified as severe.
- **Travelers visiting friends and relatives (VFR):** Immigrants, ethnically and racially distinct from the major population of the country of residence (a country where malaria is not endemic), who return to their homeland (a country where malaria is endemic) to visit friends and relatives; family members of immigrants (e.g., spouse or children) are included in this group, even if they were born in the country of residence (*37*,*38*). Non-U.S. residents who travel to visit friends and relatives in the United States also are classified as VFR travelers; however, characteristics of these persons are assessed separately from U.S. resident VFR travelers.

### Diagnosis of Malaria

Three laboratory tests can be used to diagnose malaria: 1) microscopic analysis of a peripheral blood smear (e.g., blood smear), 2) PCR, and 3) RDT; a blood smear is recommended as the preferred first-line test. If malaria is suspected, a Giemsa-stained film of the patient’s peripheral blood should be examined by microscopy for parasites as soon as possible. Microscopy allows an estimation of the level of parasitemia, which is necessary to prescribe appropriate treatment. Diagnostic accuracy depends on blood film quality and examination by experienced laboratory personnel (*39*,*40*). Three sets of thick and thin blood films spaced 12–24 hours apart are needed to rule out malaria. PCR, although not readily available to be useful in the initial diagnosis and treatment of acute malaria, is used to confirm the species, which is important for *P. vivax* and *P. ovale* infections that require additional treatment to prevent relapse (*27*,*33*).

The BinaxNOW malaria RDT (Abbott Laboratories) detects circulating malaria-specific antigens and is the only RDT approved by the Food and Drug Administration (FDA) for use by hospital and commercial laboratories; the test is not approved as a point-of-care test by clinicians or the general public (*28*,*41*). RDTs are less sensitive than microscopy and not able to determine all *Plasmodium* species or quantify malaria parasites; therefore, the results require confirmation and species identification by microscopy (*28*). If microscopy is not performed, then PCR can confirm an RDT result and determine the species.

### Drug Resistance Marker Surveillance

In 2012, CDC began molecular surveillance of imported malaria cases, with the goal of detecting and characterizing parasites that carry genetic markers (typically single nucleotide polymorphisms in one or more loci or gene copy number variation associated with antimalarial drug resistance (*42*). Molecular surveillance data are used to identify where drug-resistant foci might be present or emerging in specific parts of the world where malaria is endemic. For each sample submitted, species confirmation is conducted using a real-time PCR assay capable of detecting the four primary human-infecting *Plasmodium* species. Submitted *P. falciparum* samples are tested for molecular markers associated with resistance to sulfadoxine, pyrimethamine, chloroquine, mefloquine, atovaquone, and artemisinin. Additional markers of resistance in *P. falciparum* or other species will be similarly evaluated as they become available and as new laboratory methods are developed.

Samples for molecular resistance monitoring are processed for PCR amplification of parasite DNA using appropriate primers for the genes of interest in nested PCR assays as previously described (*43*–*47*) and sequenced by the Sanger method using the ABI 3130 capillary sequencer (Thermo Fisher Scientific). Fragments of genes encoding molecular targets of chloroquine (chloroquine resistance transporter gene, *pfcrt*), pyrimethamine and proguanil (dihydrofolate reductase gene, *pfdhfr*), sulfadoxine (dihydropteroate synthase gene, *pfdhps*), atovaquone (cytochrome b gene, *pfcytb*), mefloquine (multidrug resistance 1 protein gene, *pfmdr-1*), and artemisinin (kelch 13-propeller domain, *pfk13*) are analyzed for polymorphisms by comparing each sequence to the reference genome. The sequence data are analyzed using Geneious Pro R8 (Biomatters).

- **Chloroquine resistance markers:** The *pfcrt* gene sequence is amplified using a nested PCR method as previously described (*43*) and analyzed to identify polymorphisms C72S, M74I, N75E, and K76T.
- **Pyrimethamine and proguanil resistance markers:** The *pfdhfr* gene sequence is amplified using a nested PCR method as previously described (*43*) and analyzed to identify polymorphisms A16V, C50R, N51I, C59R, S108T/N, and I164L.
- **Sulfadoxine resistance markers:** The *pfdhps* gene sequence is amplified using a nested PCR method as previously described (*43*) and analyzed to identify polymorphisms S436A, A437G, K540E, A581G, and A631S/T.
- **Atovaquone resistance markers:** The *pfcytb* gene sequence is amplified using a nested PCR method as previously described (*44*) and analyzed to identify polymorphisms I258M and Y268S.
- **Mefloquine resistance markers:** A real-time PCR assay is used to determine the variation in the number of copies of the *pfmdr-1* gene using the comparative cycle threshold (ΔΔC_T_) method as previously described (*45*). DNA from the 3D7 laboratory control, which has a single copy of *pfmdr-1,* is used as the calibrator. In addition, DNA from Indochina W2mef and Dd2 are used as multiple copy number controls.
- **Artemisinin resistance markers:** The *pfk13* gene for artemisinin resistance is amplified using a nested PCR method as previously described (*45*,*47*) and analyzed to identify polymorphisms in the propeller domain.

## Results

### General Surveillance

**FIGURE 1:**
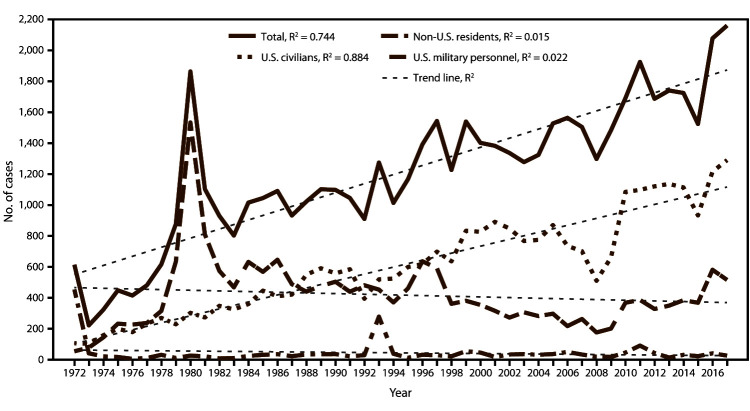
Number of malaria cases* among U.S. civilians, U.S. military personnel, and non-U.S. residents — United States, 1972–2017 **Abbreviation:** R^2^ = square of the Pearson product moment correlation coefficient. * 2017: N = 2,161.

CDC received 2,161 reports of confirmed malaria cases among persons tested in the United States and its territories, with onset of symptoms in 2017 (Table 1), representing a 4.0% relative increase in confirmed malaria cases compared with 2016 (n = 2,078). Since the United States eliminated malaria in the 1950s, a peak in the number of reported cases occurred in 1970 and 1971 (4,247 and 3,180 cases, respectively), predominantly associated with military personnel who returned from deployment in Southeast Asia; in 1972, only 614 cases were reported. Since 1972, the numbers of malaria cases have been increasing, with an average gain of 29.4 cases per year (R^2^ = 0.744) (Figure 1 and Table 1), and in 2016 and 2017, more than 2,000 cases of malaria were imported into the United States for the first time during this period. The number of malaria cases among the U.S. civilian population has increased over time, with approximately 22 cases added each year during the period 1972–2017 (R^2^ = 0.884). In contrast, the trajectory of the accumulation of military and non-U.S. cases is flat during this period (R^2^ = 0.022 and R^2^ = 0.015, respectively) (Figure 1). The increase in malaria cases coincides with the increasing trend in the annual number of international airline flights taken by U.S. citizens (*48*). Malaria diagnosed in the United States is predominantly acquired from Africa, accounting for 84.2% of all cases in 2017. The proportion of cases acquired from West Africa^†^ increased in 2017 (56.3%) compared with 2016 (51.1%). According to data from the National Travel and Tourism Office, during 1996–2017, an increase occurred in travel over time, with an average annual addition of approximately 611,000 travel events to all destinations, including an average annual gain of approximately 12,300 travel events to Africa, with a total of 403,000 travel events in 2017 from the United States to Africa (*49*).

**TABLE 1:** Number of malaria cases* among U.S. military personnel, U.S. civilians, and non-U.S residents — United States, 1970–2017

Year	U.S. military personnel	U.S. civilians	Non-U.S. residents	Status not recorded	Total
1970	4,096	90	44	17	**4,247**
1971	2,975	79	69	57	**3,180**
1972	454	106	54	0	**614**
1973	41	103	78	0	**222**
1974	21	158	144	0	**323**
1975	17	199	232	0	**448**
1976	5	178	227	5	**415**
1977	11	233	237	0	**481**
1978	31	270	315	0	**616**
1979	11	229	634	3	**877**
1980	26	303	1,534	1	**1,864**
1981	21	273	809	0	**1,103**
1982	8	348	574	0	**930**
1983	10	325	468	0	**803**
1984	24	360	632	0	**1,016**
1985	31	446	568	0	**1,045**
1986	35	410	646	0	**1,091**
1987	23	421	488	0	**932**
1988	33	550	440	0	**1,023**
1989	35	591	476	0	**1,102**
1990	36	558	504	0	**1,098**
1991	22	585	439	0	**1,046**
1992	29	394	481	6	**910**
1993	278	519	453	25	**1,275**
1994	38	524	370	82	**1,014**
1995	12	599	461	95	**1,167**
1996	32	618	636	106	**1,392**
1997	28	698	592	226	**1,544**
1998	22	636	361	208	**1,227**
1999	55	833	381	271	**1,540**
2000	46	827	354	175	**1,402**
2001	18	891	316	158	**1,383**
2002	33	849	272	183	**1,337**
2003	36	767	306	169	**1,278**
2004	32	775	282	235	**1,324**
2005	36	870	297	325	**1,528**
2006	50	736	217	561	**1,564**
2007	33	701	263	508	**1,505**
2008	19	510	176	593	**1,298**
2009	18	661	201	604	**1,484**
2010	46	1,085	368	192	**1,691**
2011	91	1,098	386	350	**1,925**
2012	43	1,121	328	195	**1,687**
2013	14	1,136	349	242	**1,741**
2014	31	1,114	384	196	**1,725**
2015	23	933	368	200	**1,524**
2016	41	1,216	581	240	**2,078**
2017	26	1,290	516	329	**2,161**

* A case was defined as symptomatic or asymptomatic illness that occurs in the United States or one of its territories in a person who has laboratory-confirmed malaria parasitemia (microscopy or polymerase chain reaction), regardless of whether the person had previous attacks of malaria while in other countries. A subsequent attack of malaria occurring in a person is counted as an additional case, unless it occurred as a result of a drug resistance failure. Relapsing illnesses are counted as a subsequent case.

In 2017, a total of 1,290 (59.7%) cases occurred among U.S. civilians, 26 (1.2%) among U.S. military members, 516 (23.9%) among non-U.S. residents, and 329 (15.2%) among patients with unknown or unreported resident status (Table 1). Of the 2,161 confirmed cases, 2,112 cases were imported from countries in which malaria is endemic; two cases were classified as congenital, three remained classified as cryptic after an investigation, and two were transmitted through a blood transfusion. A total of 42 case reports had an incomplete travel history that prevented classification. Of the 42 cases with unknown classification, four had laboratory reports only, and 38 were reported by local or state health departments as lost to follow-up with insufficient information to classify importation status. In 2017, a total of 25.6% of case reports were missing one or more of the essential variables (infecting species, residence status, and country of acquisition); overall, these variables were less complete in 2017 than in 2016 (20.6%). In 2017, among all cases, 10.5% were missing the infecting species (versus 10.8% in 2016), 15.2% were missing residence status (versus 11.5% in 2016), and 4.0% were missing the country of acquisition (versus 3.0% in 2016). No case in 2017 was reported as having been acquired by mosquitoborne transmission in the United States.

### *Plasmodium* Species

In 2017, a total of 1,935 (89.5%) cases reported to CDC included information on the infecting *Plasmodium* species (Table 2), which is similar to the proportion of cases in 2016 (89.2%). Specimens from 206 (9.5%) cases were sent to the CDC reference laboratory for diagnostic assistance, and CDC identified the species for 202 (98.1%) specimens. An additional 153 persons had specimens submitted for malaria testing, and all were negative for *Plasmodium* parasites.

**TABLE 2:** Number of malaria cases, by *Plasmodium* species and year — United States, 2013–2017

*Plasmodium* species	2013			2014			2015			2016			2017		
	No.	(%)*	(%)^†^	No.	(%)*	(%)^†^	No.	(%)*	(%)^†^	No.	(%)*	(%)^†^	No.	(%)*	(%)^†^
*P. falciparum*	1,059	(60.8)	(72.8)	1,141	(66.1)	(74.9)	1,025	(67.3)	(77.2)	1,419	(68.3)	(76.6)	1,523	(70.5)	(78.7)
*P. vivax*	245	(14.1)	(16.8)	230	(13.3)	(15.1)	180	(11.8)	(13.6)	251	(12.1)	(13.6)	216	(10.0)	(11.2)
*P. ovale*	65	(3.7)	(4.5)	90	(5.2)	(5.9)	63	(4.1)	(4.7)	99	(4.8)	(5.3)	119	(5.5)	(6.2)
*P. malariae*	45	(2.6)	(3.1)	47	(2.7)	(3.1)	48	(3.2)	(3.6)	61	(2.9)	(3.3)	55	(2.6)	(2.8)
Mixed	41	(2.4)	(2.8)	15	(0.9)	(1.0)	12	(0.8)	(0.9)	23	(1.1)	(1.2)	22	(1.0)	(1.1)
Undetermined	286	(16.4)	—	202	(11.7)	—	196	(12.9)	—	225	(10.8)	—	226	(10.5)	—
**Total**	**1,741**	**(100)**	**(100)**	**1,725**	**(100)**	**(100)**	**1,524**	**(100)**	**(100)**	**2,078**	**(100)**	**(100)**	**2,161**	**(100)**	**(100)**

* Percentage among all infections.

^†^ Percentage among infections with known species.

Among the 1,935 cases with a species determination, 1,523 (78.7%) were *P. falciparum* (Table 2). During 2012–2017, there was an increasing proportion of *P. falciparum* cases with an average addition of 104.4 cases of this species each year, a percent increase of 54.8%. In 2017, there were 216 (11.2%) *P. vivax* infections. During 2012–2017, there was a decreasing proportion of *P. vivax* cases, with an average loss of 10.1 each year. In 2017, a total of 119 (6.2%) *P. ovale*, 55 (2.8%) *P. malariae*, and 22 (1.1%) mixed-species infections were identified. Of the 22 mixed infections, nine were *P. falciparum* with *P. malariae*, five were *P. falciparum* with *P. vivax*, four were *P. malariae* with *P. ovale*, three were *P. falciparum* with *P. ovale*, and one was *P. falciparum*, *P. ovale*, and *P. malariae.* Of 22 mixed infections, 19 were PCR confirmed, seven of these by the CDC reference laboratory, including the specimen positive for three *Plasmodium* species. The species distribution in 2017 for *P. falciparum*, *P. vivax*, *P. ovale*, *P. malariae*¸ mixed *Plasmodium* species, and unknown species infections was similar to the 2016 distribution. During 2012–2017, the proportion of unspeciated cases decreased, with a 20.8% decrease in cases per year. Because of the small number of *P. ovale*, *P. malariae,* and mixed-species infections detected each year, proportional changes over time were not assessed for these infections.

In 2017, a total of 764 (35.4%) malaria infections reported in the United States were confirmed by PCR, a similar proportion to 2016, when 717 of 2,078 (34.5%) infections were PCR confirmed. Approximately one third of *P. falciparum* and *P. vivax* infections were PCR confirmed (568 [37.3%] and 76 [35.2%], respectively). Of 55 *P. malariae* cases, 28 (50.9%) were PCR confirmed; 55 (46.2%) of 119 *P. ovale* infections were PCR confirmed, and 19 (86.4%) of 22 mixed species infections were PCR confirmed. Of 226 unspeciated infections, 18 (8.0%) were PCR confirmed to the *Plasmodium* genus only.

### Region of Acquisition and Diagnosis

Information on the region of acquisition was available for 2,073 (98.2%) of 2,112 imported cases; of these, 1,819 (87.8%) were from Africa, 181 (8.7%) from Asia, 37 (1.8%) from South America, 30 (1.5%) from Central America and the Caribbean, and six (<1.0%) from Oceania (Table 3).

**TABLE 3:** Number of imported malaria cases (including PCR-confirmed cases), by country of acquisition and *Plasmodium* species — United States, 2017

Country of acquisition	*P. falciparum*		*P. vivax*		*P. ovale*		*P. malariae*		Mixed		Unknown		Total	
	PCR	Total	PCR	Total	PCR	Total	PCR	Total	PCR	Total	PCR	Total	PCR	Total
**Africa**	**434**	**1,435**	**9**	**34**	**45**	**113**	**19**	**48**	**15**	**20**	**14**	**169**	**536**	**1,819**
Angola	3	10	0	0	0	1	0	0	0	0	0	1	**3**	**12**
Benin	4	7	0	0	0	0	0	0	0	0	0	0	**4**	**7**
Burkina Faso	14	24	0	0	0	2	0	0	0	0	0	0	**14**	**26**
Burundi	4	6	1	1	0	1	0	0	0	0	0	0	**5**	**8**
Cameroon	27	111	0	1	8	17	0	4	1	2	0	12	**36**	**147**
Central African Republic	0	6	0	0	0	0	0	0	1	1	0	0	**1**	**7**
Chad	2	3	0	0	0	0	0	0	0	0	0	0	**2**	**3**
Congo, Democratic Republic of	6	29	1	1	3	4	1	1	4	4	0	9	**15**	**48**
Djibouti	0	0	1	2	0	0	0	0	0	0	0	0	**1**	**2**
Equatorial Guinea	1	4	0	0	0	1	0	0	0	0	0	0	**1**	**5**
Eritrea	0	2	0	1	0	0	0	0	0	0	0	1	**0**	**4**
Ethiopia	2	6	2	7	0	1	0	1	1	2	0	4	**5**	**21**
Gabon	0	1	0	0	0	0	0	0	0	0	1	1	**1**	**2**
Gambia, The	2	2	0	0	0	0	0	0	0	0	0	1	**2**	**3**
Ghana	44	123	0	0	2	4	1	1	0	0	2	17	**49**	**145**
Guinea	26	53	0	0	3	4	1	1	1	1	1	1	**32**	**60**
Guinea-Bissau	2	2	0	0	0	0	0	0	1	1	0	0	**3**	**3**
Ivory Coast	30	75	0	1	3	6	1	3	1	1	0	3	**35**	**89**
Kenya	13	47	1	3	0	4	1	5	0	0	1	4	**16**	**63**
Liberia	18	108	0	2	0	0	4	5	0	0	1	18	**23**	**133**
Malawi	1	7	0	0	0	1	1	1	0	0	1	2	**3**	**11**
Mali	3	10	0	0	0	0	0	1	0	0	0	1	**3**	**12**
Mozambique	3	9	0	0	1	1	0	0	0	0	0	2	**4**	**12**
Niger	0	2	0	0	0	0	0	1	0	0	0	1	**0**	**4**
Nigeria	135	415	1	3	12	30	2	4	0	0	3	49	**153**	**501**
Rwanda	2	9	0	1	3	5	1	3	0	0	0	2	**6**	**20**
Senegal	3	9	0	0	0	1	0	0	0	0	0	0	**3**	**10**
Sierra Leone	32	134	0	1	2	10	1	2	0	0	1	14	**36**	**161**
Somali Republic	1	1	0	1	0	0	0	0	1	1	0	1	**2**	**4**
South Africa	1	2	0	0	0	0	0	0	0	0	0	1	**1**	**3**
South Sudan	1	6	0	0	1	1	0	0	0	0	0	1	**2**	**8**
Sudan	12	28	1	3	1	1	1	2	0	1	0	1	**15**	**36**
Tanzania	3	16	0	0	4	6	1	2	0	0	0	3	**8**	**27**
Togo	7	34	1	1	0	1	0	0	0	0	0	0	**8**	**36**
Uganda	12	49	0	3	2	8	1	8	2	4	2	11	**19**	**83**
Zambia	1	4	0	0	0	1	0	1	0	0	0	0	**1**	**6**
Zimbabwe	1	5	0	0	0	0	0	0	0	0	0	0	**1**	**5**
Africa, unspecified	6	26	0	1	0	1	2	2	1	1	1	7	**10**	**38**
Central Africa, unspecified	0	2	0	0	0	0	0	0	0	0	0	0	**0**	**2**
East Africa, unspecified	4	18	0	0	0	0	0	0	0	0	0	1	**4**	**19**
South Africa, unspecified	0	7	0	0	0	0	0	0	0	0	0	0	**0**	**7**
West Africa, unspecified	8	23	0	1	0	1	0	0	1	1	0	0	**9**	**26**
**Asia**	**1**	**13**	**40**	**132**	**1**	**1**	**0**	**1**	**1**	**2**	**2**	**32**	**44**	**181**
Afghanistan	0	1	10	36	0	0	0	0	0	0	1	11	**11**	**48**
Cambodia	0	0	1	1	0	0	0	0	0	0	0	0	**1**	**1**
India	1	10	21	76	1	1	0	1	1	1	0	16	**23**	**105**
Indonesia	0	0	0	1	0	0	0	0	0	1	0	1	**0**	**3**
Malaysia	0	0	1	1	0	0	0	0	0	0	0	0	**1**	**1**
Pakistan	0	1	6	14	0	0	0	0	0	0	1	3	**7**	**18**
Southeast Asia, unspecified	0	1	1	2	0	0	0	0	0	0	0	1	**1**	**4**
Southern Asia, unspecified	0	0	0	1	0	0	0	0	0	0	0	0	**0**	**1**
**Central America and the Caribbean**	**4**	**17**	**3**	**9**	**0**	**0**	**0**	**1**	**0**	**0**	**0**	**3**	**7**	**30**
Dominican Republic	0	4	0	0	0	0	0	0	0	0	0	0	**0**	**4**
Guatemala	0	0	0	3	0	0	0	0	0	0	0	2	**0**	**5**
Haiti	3	12	0	0	0	0	0	1	0	0	0	0	**3**	**13**
Honduras	0	0	2	3	0	0	0	0	0	0	0	1	**2**	**4**
Nicaragua	1	1	0	0	0	0	0	0	0	0	0	0	**1**	**1**
Central America, unspecified	0	0	1	3	0	0	0	0	0	0	0	0	**1**	**3**
**South America**	**3**	**6**	**14**	**29**	**0**	**0**	**0**	**0**	**0**	**0**	**0**	**2**	**17**	**37**
Brazil	0	1	2	5	0	0	0	0	0	0	0	0	**2**	**6**
Colombia	0	1	0	1	0	0	0	0	0	0	0	0	**0**	**2**
Guyana	3	4	11	13	0	0	0	0	0	0	0	0	**14**	**17**
Peru	0	0	1	5	0	0	0	0	0	0	0	2	**1**	**7**
Venezuela	0	0	0	3	0	0	0	0	0	0	0	0	**0**	**3**
South America, unspecified	0	0	0	2	0	0	0	0	0	0	0	0	**0**	**2**
**Oceania**	**1**	**1**	**1**	**4**	**0**	**0**	**0**	**0**	**0**	**0**	**0**	**1**	**2**	**6**
Papua New Guinea	1	1	1	4	0	0	0	0	0	0	0	1	**2**	**6**
**Unknown**	**9**	**22**	**0**	**4**	**0**	**0**	**1**	**2**	**0**	**0**	**1**	**11**	**11**	**39**
**Total**	**452**	**1,494**	**67**	**212**	**46**	**114**	**20**	**52**	**16**	**22**	**17**	**218**	**618**	**2,112**

**Abbreviation:** PCR = polymerase chain reaction.

Of the 1,472 imported *P. falciparum* cases reported in 2017 with a known region of acquisition, 1,435 (97.5%) were acquired from Africa, 17 (1.2%) from Central America and the Caribbean, 13 (0.9%) from Asia, six (<1.0%) from South America, and one from Oceania (<1.0%). *P. falciparum* infections accounted for 78.9% of the cases imported from Africa, 7.2% from Asia, 56.7% from Central America and the Caribbean, 16.2% from South America, and 16.7% from Oceania (Table 3).

In 2017, a total of 208 imported *P. vivax* infections were reported with information about region of acquisition. Of these, 132 (63.5%) were acquired from Asia, followed by 34 (16.4%) from Africa, 29 (13.9%) from South America, nine (4.3%) from Central America and the Caribbean, and four (1.9%) from Oceania. In 2017, a total of 114 *P. ovale* and 50 *P. malariae* infections were reported with information about the region of acquisition; 113 (99.1%) and 48 (96.0%) were acquired from Africa, respectively. The geographical area of acquisition was reported for all 22 mixed-species infections; 20 (90.9%) mixed-species infections were acquired from Africa and two (9.1%) from Asia (Table 3).

In 2017, among imported cases with a known region of acquisition, no difference was found in the proportion of malaria cases from Africa as compared with 2016 (1,819 [87.8%] of 2,073 cases in 2017, compared with 1,729 [85.8%] of 2,016 cases in 2016). However, in 2017, the proportion of cases imported from West Africa increased; 1,216 (57.6%) of all cases were imported from West Africa in 2017, compared with 1,061 (51.6%) in 2016. Of the five countries most commonly reported as the country of acquisition in 2017, four were in West Africa (Nigeria, 501 cases; Sierra Leone, 161 cases; Ghana, 145 cases; and Liberia, 133 cases). A total of 147 persons had traveled to Cameroon, in Central Africa, in 2017. Compared with 2016, an increase of at least 20 additional cases were imported from the following African countries in 2017: Nigeria (55 more), Liberia (36 more), Ivory Coast (30 more), Sierra Leone (27 more), Cameroon (25 more), and Ghana (20 more). Kenya, Togo, and Zimbabwe all had five to nine more cases in 2017 than in 2016. Countries in Africa with fewer cases imported into the United States in 2017 than in 2016 included Uganda (40 fewer), Democratic Republic of Congo (30 fewer), Tanzania (23 fewer), Ethiopia (20 fewer), Chad (12 fewer), and Burkina Faso (10 fewer). Niger, Senegal, Gabon, and South Sudan all had five to nine fewer cases imported in 2017 than in 2016.

The number and proportion of cases imported from Asia were similar in 2017 compared with 2016 (181 [8.6%] and 174 [8.6%], respectively). The greatest number of cases imported from this region were from India, with 105 cases in 2017 and 89 cases in 2016. Cases imported from Afghanistan increased from 39 in 2016 to 48 in 2017. No cases were acquired from South Korea in 2017, compared with 10 in 2016.

The proportion of imported cases from Central America and the Caribbean decreased from 70 cases in 2016 to 30 cases in 2017. Among the seven countries in Central America and the Caribbean from which cases of malaria were acquired in 2016 and 2017, the number of cases decreased in six (Haiti, the Dominican Republic, Honduras, Guatemala, Panama, and Mexico), and one case was reported from Nicaragua in both 2016 and 2017. In 2017, fewer than half as many cases were reported from the Central American region (35 cases in 2016 and 13 cases in 2017) and from the Caribbean region (35 cases in 2016 and 17 cases in 2017).

Of the 17 cases from the Caribbean region (Haiti and the Dominican Republic), 16 (94.1%) were *P. falciparum* species (Table 3). The number of cases from Haiti decreased from a peak of 171 cases in 2010, to 22 in 2016, and to 13 in 2017; two of 13 patients had severe illness. During 2005–2015, the United States had an average of 4.6 cases per year imported from the Dominican Republic. During 2014, two cases were imported from the Dominican Republic. During 2015, the number increased significantly to 32 cases, and included eight cases imported into Puerto Rico (*48*,*50*); the cases acquired from the Dominican Republic were predominantly among tourists. In 2016, a total of 13 cases were imported from the Dominican Republic, compared with four cases in 2017. Of the four patients who had traveled to the Dominican Republic, three had traveled for tourism in 2017, and one did not provide a reason for travel. Of the four cases from the Dominican Republic, one patient had severe malaria.

Of 13 cases from Central America, nine were *P. vivax*, one was *P. falciparum*, and three were unspecified species. Among cases acquired from Central America, one severe case (of unknown species) was acquired from Guatemala. Of persons with malaria who had traveled from Central America, six were refugees or immigrants, three had traveled for tourism, two had traveled as missionaries, and two had traveled to visit friends and family.

During both 2016 and 2017, a total of 37 cases were imported from South America each year. In both years, Guyana, Peru, and Brazil were the sources of the majority of the cases acquired in South America. In 2017, a total of 17 cases from Guyana, seven from Peru, and six from Brazil were reported. Of 37 infections acquired in South America, 29 were *P. vivax*, six were *P. falciparum*, and two did not have the infecting species determined; two patients, both with travel to Guyana, had severe *P. vivax* infection. VFR travel and tourism were reported as reasons for travel by nine persons each, two persons traveled as missionaries, and 17 persons had unknown or other reasons for travel to South America.

As in 2016, six cases were reported from Oceania in 2017, all of them from Papua New Guinea; four of six cases were *P. vivax*, one was *P. falciparum,* and one was reported with the species not determined. None of these patients had severe illness. Of the six patients, two had traveled as missionaries, and one each traveled to visit friends and family, for business, for education (as a teacher or student), and for an unknown reason.

**FIGURE 2:**
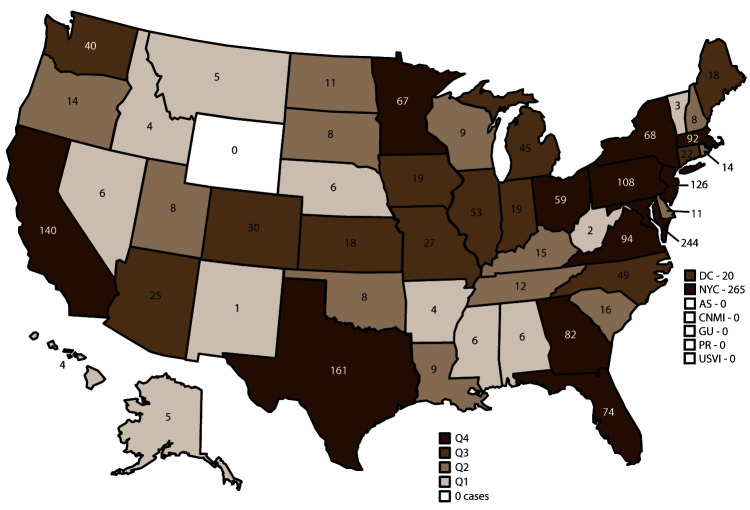
Number of malaria cases,* by state and quartile^†^ — United States, 2017 **Abbreviations:** AS = American Samoa; CNMI = Commonwealth of the Northern Mariana Islands; DC = Washington, DC; GU = Guam; NYC = New York City; PR = Puerto Rico; Q = quartile; USVI = U.S. Virgin Islands. * N = 2,161. New York state cases do not include those from New York City. ^†^ States reporting one or more cases in 2017 were categorized into quartiles: Q1 = 1–7 cases (12 states); Q2 = 8–17 cases (13 states); Q3 = 18–58 cases (13 states); and Q4 = 59 or more cases (13 states).

Confirmed cases were classified according to location of diagnosis or residence of the infected person. In 2017, Wyoming was the only state that did not report at least one case of malaria. States reporting one or more cases were categorized into quartiles (Figure 2); the 13 jurisdictions in the upper quartile accounted for 73.1% of the 2,161 cases reported. These included New York City (265), Maryland (244), Texas (161), California (140), New Jersey (126), Pennsylvania (108), Massachusetts (92), Virginia (94), Florida (74), Georgia (82), New York State (not including New York City) (68), Minnesota (67), and Ohio (59). Compared with 2016, a total of 10 jurisdictions reported an increase of five or more cases, and 32 reported no change or a decrease in cases. Two jurisdictions reported an increase of at least 40 cases in 2017 compared with 2016: Maryland (62 more) and New Jersey (40 more). Four jurisdictions reported an increase of at least 10 or more cases in 2017 compared with 2016: Pennsylvania (24 more), Virginia (19 more), California (15 more), and Georgia (13 more). The states with the most substantial decrease in malaria cases in 2017 were Arizona (15 fewer), Tennessee (14 fewer), Wisconsin (12 fewer), and Illinois (12 fewer).

### Imported Malaria Among U.S. Residents and Nonresidents

Among 2,112 imported cases, information on residence status was available for 1,823 (86.3%) cases; of these, 1,308 (71.8%) persons were U.S. residents (civilian and military) and 515 (28.3%) were non-U.S. residents (Table 4). In 2017, fewer non-U.S. residents received diagnoses of malaria compared with 2016 (515 [28.2%] cases in 2017 versus 581 [31.7%] in 2016). The majority of infections among U.S. residents were acquired from Africa (1,181 [90.3%]); this represented an increase compared with 2016 (1,087 [86.8%]) and continued an upward trend observed since 2008 (*11*,*13*–*15*,*42*,*48*,*51*–*53*). Fewer U.S. residents acquired malaria from Asia (59 [4.5%] in 2017 versus 88 [7.1%] in 2016) and the Caribbean (12 [0.9%] in 2017 versus 28 [2.3%] in 2016). No significant change occurred from 2016 to 2017 in the number of U.S. residents with imported malaria infections from West Africa (806 [61.6%] in 2017 versus 739 [59.0%] in 2016), Central America (6 [0.5%] in 2017 versus 11 [0.9%] in 2016), or South America (21 [1.6%] in 2017 versus 23 [1.9%] in 2016). Among U.S. residents, four cases (<1.0%) were acquired from Oceania each year in 2016 and 2017.

**TABLE 4:** Number and percentage of imported malaria cases among U.S residents and non-U.S. residents, by region of acquisition — United States, 2017

Area or region	U.S. residents	Non-U.S. residents	Total
	No. (%)	No. (%)	No. (%)
Africa	1,181 (90.3)	397 (77.1)	**1,578 (86.6)**
Asia	59 (4.5)	89 (17.3)	**148 (8.1)**
South America	21 (1.6)	13 (2.5)	**34 (1.9)**
Central America/Caribbean	18 (1.4)	10 (1.9)	**28 (1.5)**
Oceania	4 (0.3)	2 (0.4)	**6 (0.3)**
Unknown	25 (1.9)	4 (0.8)	**29 (1.6)**
**Total**	**1,308 (100)**	**515 (100)**	**1,823 (100)**

Most malaria cases imported among the 515 non-U.S. residents in 2017 were from Africa (397 [77.1%]), followed by Asia (89 [17.3%]) and the Americas and Caribbean (23 [4.5%]) (Table 4). Among non-U.S. residents, a similar proportion of cases was imported from Africa in 2017 and 2016 (77.1% in 2017 versus 81.6% in 2016), although the proportion from West Africa increased in 2017 (234 [45.4%] cases in 2017 versus 227 [39.1%] in 2016). The proportion of non-U.S. residents who had acquired malaria from Asia increased in 2017 (89 [17.3%] cases in 2017 versus 70 [12.1%]). The number of non-U.S. resident malaria patients who traveled to the United States in 2017 from South and Central America and the Caribbean regions was low (23 [4.5%] total), and this proportion was similar to that in 2016 (35 [6.0%] cases). The number of non-U.S. residents who traveled from Oceania was two in 2017 and none in 2016. From 2016 to 2017, ≥10 more cases were reported among residents of Sierra Leone (28 cases in 2017 and 11 cases in 2016), Afghanistan (39 cases in 2017 and 24 cases in 2016), Ghana (24 cases in 2017 and 11 cases in 2016), and India (41 cases in 2017 and 31 cases in 2016). Reductions of ≥10 fewer cases in 2017 were reported among residents from Tanzania (nine in 2017 and 34 in 2016), Democratic Republic of Congo (30 in 2017 and 50 in 2016), and Burkina Faso (six in 2017 and 16 in 2016).

In 2016, after a decrease in imported malaria cases acquired from West Africa in 2015 because of the Ebola virus disease outbreak and travel restrictions (*48*,*54*–*57*), malaria cases increased among U.S. residents and decreased among non-U.S. residents who acquired malaria from previously Ebola-affected countries (*13*). In 2017, presumably because of continued travel to and from these countries, the high level of cases acquired among U.S. residents from previously Ebola-affected countries was comparable to what was observed in 2016 (235 [18.0%] cases in 2017 versus 224 [17.9%] cases in 2016). In 2017, among non-U.S. residents, an increase in malaria cases acquired from Ebola-affected countries occurred (64 [12.4%] cases in 2017 versus 42 [7.2%] cases in 2016), predominantly among residents from Sierra Leone (17 more cases) and Liberia (seven more cases), whereas Guinea had two fewer cases in 2017 than in 2016.

### Seasonality of Malaria Diagnosed in the United States

**FIGURE 3:**
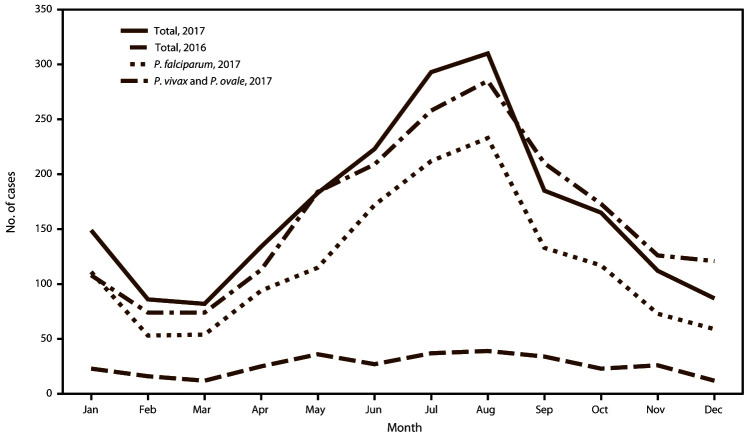
Number* of imported malaria cases, by *Plasmodium* species and month of symptom onset — United States, 2016^†^ and 2017^§^ * 2016: N = 1,935; 2017: N = 2,009. ^†^ 2016: Information about the month of illness onset was available for 1,935 (94.1%) of 2,056 imported cases. ^§^ 2017: Information about the month of illness onset was available for 2,009 (95.1%) of 2,112 imported cases.

In 2017, the peak of travel for U.S. citizens to Africa occurred in the summer months, with a total of approximately 44,000 flight departures in June and 46,000 in July. In August, approximately 40,000 departures were reported (*49*). The mean number of flight departures for 2017 was approximately 33,500 per month. A total of 36,500 flight departures occurred in December 2017 (*49*). As in previous years (*11*,*13*–*15*,*42*,*48*,*51*–*53*), imported malaria cases peaked during the months of July and August 2017, coinciding with summer travel. Information about the month of illness onset was available for 2,009 (95.1%) of 2,112 imported cases. Overall, the mean number of imported cases per month was 167.4; an average of 301.5 cases occurred in July and August, with 293 cases in July and 310 cases in August 2017 (Figure 3). The lowest numbers of malaria cases were reported in February, March, and December, with 86, 82, and 87 cases, respectively. In 2017, the months with the greatest increase compared with 2016 were January (41 more), July (35 more), and August (25 more). September and December 2017 had 25 and 34 fewer cases than for the same month in 2016, respectively. *P. falciparum* accounted for 444 (73.6%) of 603 cases imported in July and August 2017 and 166 (65.1%) of 255 cases in February, March, and December 2017. *P. vivax* and *P. ovale* infections were combined because the illnesses can have a delayed initial onset after infection or can result from a relapse, which might show as a secondary peak in the timeline of infections. In 2017, the mean number of *P. vivax* and *P. ovale* cases was 25.8 per month; the maximum numbers occurred in July and August (37 and 39 cases per month, respectively). Monthly numbers of *P. vivax* and *P. ovale* cases were above the mean during May–September.

### Interval Between Arrival in the United States and Illness Onset

Among 1,894 imported cases with a *Plasmodium* species determination, 1,475 (77.9%) had complete information on return travel and illness onset dates, allowing for calculation of the interval between these dates (Table 5). Among these patients, regardless of infecting species, 177 (12.0%) had symptom onset before arriving in the United States. Among 1,203 patients with *P. falciparum* infections, 1,160 (96.4%) had symptom onset before or within 29 days of arrival in the United States. In contrast, 58 (40.6%) of 143 *P. vivax* patients and 42 (52.5%) of 80 *P. ovale* patients had illness onset ≥30 days after arrival in the United States, consistent with the potential for these species to relapse because of the persistence of liver hypnozoites or to have an extended incubation period before symptom onset (*3*–*5*). Of infections with any species, 99% occurred within 1 year of arrival in the United States after travel to a country where malaria is endemic. Of the nine persons with illness onset ≥1 year after reported travel, eight of the infections were *P. vivax* or *P. ovale* species; one person, an immigrant aged 17 years from Ghana, had a PCR-confirmed *P. falciparum* illness ≥1 year after arrival in the United States.

**TABLE 5:** Number and percentage of imported malaria cases, by *Plasmodium* species* and interval between date of arrival in the United States and onset of illness — United States, 2017

Interval (days)	*P. falciparum*	*P. vivax*	*P. ovale*	*P. malariae*	Mixed	Total
	No. (%)	No. (%)	No. (%)	No. (%)	No. (%)	No. (%)
<0^†^	159 (13.2)	10 (7.0)	4 (5.0)	0 (0)	4 (22.2)	**177 (12.0)**
0–29	1,001 (83.2)	75 (52.5)	34 (42.5)	17 (54.8)	7 (38.9)	**1,134 (76.9)**
30–89	35 (2.9)	22 (15.4)	16 (20.0)	11 (35.5)	4 (22.2)	**88 (6.0)**
90–179	4 (0.3)	19 (13.3)	9 (11.3)	2 (6.5)	3 (16.7)	**37 (2.5)**
180–364	3 (0.3)	15 (10.5)	11 (13.8)	1 (3.2)	0 (0)	**30 (2.0)**
≥365	1 (0.1)	2 (1.4)	6 (7.5)	0 (0)	0 (0)	**9 (0.6)**
**Total**	**1,203 (100.0)**	**143 (100.0)**	**80 (100.0)**	**31 (100.0)**	**18 (100.0)**	**1,475 (100.0)**

* Persons for whom *Plasmodium* species, date of arrival in the United States, or date of onset of illness is unknown are not included.

^†^ Cases in this row are among patients who had onset of illness before arriving in the United States.

### Imported Malaria Among U.S. Military Personnel

In 2017, a total of 26 cases of malaria diagnosed in the United States occurred among U.S. military personnel, a decrease compared with 41 cases reported in 2016 (1.2% and 2.0% of imported cases in 2017 and 2016, respectively). In 2017, a total of 20 cases of malaria among U.S. military members were acquired from Africa, three cases were acquired from Asia, and for three cases the country or region of acquisition was unknown. Among cases acquired from Africa, five were acquired among service personnel traveling to Cameroon; four to Nigeria; two each to Liberia, Sierra Leone, and unspecified Africa; and one each to Ghana, Ivory Coast, Tanzania, Togo, and Uganda. The regional proportions of where malaria was acquired by U.S. military members were different in 2017 compared with 2016. In 2016, equal numbers of cases were acquired from Africa and Asia (17 cases [41.5%] each). In 2017, a total of 20 (76.9%) cases were acquired from Africa, and three (11.5%) were from Asia. A total of 11 (42.3%) cases were from West Africa in 2017, whereas five (12.2%) cases were from West Africa in 2016. No cases of malaria reported among service members from South Korea in 2017, whereas 10 cases were reported in 2016. In 2017, three cases were acquired from Afghanistan, compared with seven in 2016. Malaria was acquired by six military personnel during personal leave each year in 2016 and 2017. The *Plasmodium* species was known for 22 (84.6%) of 26 cases among military personnel diagnosed in the United States in 2017. Of these, 12 (54.6%) were *P. falciparum*, five (22.7%) were *P. ovale*, three (13.6%) were *P. vivax,* and two (9.1%) were *P. malariae*. Of 26 military cases, seven were PCR confirmed (four *P. falciparum*, two *P. ovale*, and one *P. malariae*).

Reports for 24 (92.3%) military patients had information on chemoprophylaxis use; 11 did not take an antimalarial medication to prevent malaria. Of the 10 patients who took chemoprophylaxis and whose region of travel was known, nine took an appropriate regimen for the region of travel: six took doxycycline, two took atovaquone-proguanil, and one patient took two or more medications for chemoprophylaxis. The chemoprophylaxis medication was not specified for three patients. Of seven patients with information on chemoprophylaxis adherence, five reported missing doses. Of the 19 military members who had traveled to Africa and had information available on chemoprophylaxis use, nine (47.4%) reported taking chemoprophylaxis. Of three military patients who had traveled to Asia, two (66.7%)reported taking chemoprophylaxis. Of eight military patients with *P. vivax* or *P. ovale* diagnoses, three reported taking primaquine to prevent a relapse.

Among military patients in the United States, four received a diagnosis of severe malaria; all had traveled to Africa and were infected with *P. falciparum*. Of these four patients, three had documented hyperparasitemia (≥5% red blood cells infected), and three were treated with an intravenous antimalarial (e.g., artesunate or quinidine); one patient with 9.2% parasitemia had traveled to visit friends and family while on leave from the military and after receiving the diagnosis was treated with an oral regimen of atovaquone-proguanil; the repeat blood smear was negative. None of the four military patients with severe malaria was reported to have died from this illness.

### Chemoprophylaxis Use Among U.S. Residents

Among U.S. residents (civilian and military), information about malaria chemoprophylaxis use was reported for 1,157 (88.5%) of 1,308 imported cases; among these patients, 829 (71.7%) indicated that no chemoprophylaxis medication was taken during travel. Among the 328 patients who reported taking chemoprophylaxis, 115 (35.1%) did not indicate which medication was taken. Among 213 patients who reported specific drug information, 180 (84.5%) took a regimen recommended by CDC for the region of travel. Among 157 reports containing self-reported adherence information, 90 (57.3%) patients reported missing doses. Of the 90 U.S. residents with malaria who initiated an appropriate chemoprophylaxis regimen but did not adhere to it entirely, 75 (83.3%) persons provided reasons for missing doses, which included prematurely stopping after leaving the area where malaria was endemic (30.7%), forgetting to take the medication (28.0%), stopping because of a side effect (12.0%), and stopping because they didn’t think it was needed (12.0%); unknown and other reasons (e.g., lost or stolen medication and being advised to stop) also were reported, although less frequently. Although not formally asked about on the malaria case report form, 12% of respondents answered that they stopped prophylaxis because of a prescription error or running out of the medication. Of the nine persons who reported stopping prophylaxis because of side effects, eight took doxycycline; of those, four persons reported gastrointestinal side effects, one person reported discoloration of the skin, one reported nightmares and depression, and two did not specify a side effect. In addition, one patient who took atovaquone-proguanil for prevention stopped because of symptoms of fatigue. Among patients who took chemoprophylaxis, reported adherence was comparable in 2017 to adherence in 2016 (67 [42.7%] of 157 in 2017 versus 58 [43.9%] of 132 in 2016). Among 180 patients who took CDC-recommended chemoprophylaxis, 68 (37.8%) took doxycycline, 58 (32.2%) took mefloquine, 45 (25.0%) took atovaquone-proguanil, three (1.7%) took chloroquine, and six (3.3%) took two or more CDC-recommended medications. Of the 717 U.S. residents classified as VFR travelers and for whom information on chemoprophylaxis use was available, 173 (24.1%) took any prophylaxis, which was lower than among non-VFR travelers (155 [35.2%]). In addition, a lower proportion of VFR travelers (30 [4.8%] of 622) reported completing the regimen of chemoprophylaxis compared with non-VFR travelers (37 [9.9%] of 373). Altogether, among the 995 U.S. resident cases with complete information on chemoprophylaxis, 67 (6.7%) patients were adherent to an appropriate regimen, 838 (84.2%) patients did not take chemoprophylaxis or took an incorrect regimen, and 90 (9.1%) patients took an appropriate regimen but reported incomplete adherence. Thus, 93% (928 of 995) of U.S. residents with malaria either did not take or did not adhere to a CDC-recommended chemoprophylaxis regimen.

#### Cases of *P. vivax* or *P. ovale* Infections After CDC-Recommended Prophylaxis Use

The infecting malaria species was known for 162 (90.0%) of 180 patients who took a CDC-recommended chemoprophylaxis regimen; *P. vivax* accounted for 14 (8.6%) and *P. ovale* for 15 (9.3%) of these cases. Primary prophylaxis can prevent acute illness in *P. vivax* and *P. ovale* infections, although the patient might experience a relapsing illness unless primaquine is taken to eliminate dormant hypnozoites (radical cure) (*33*). A previous history of malaria or illness onset >45 days after return to the United States is consistent with relapsing illnesses. Of 29 patients with *P. vivax* or *P. ovale* infections who took chemoprophylaxis, two reported a previous history of malaria within the past 12 months. Sufficient information was available for 20 of 29 patients with *P. vivax* or *P. ovale* infections to calculate the number of days between the return travel date and the date of illness onset; symptom onset occurred >45 days after return to the United States among 12 patients. Of the eight cases that occurred ≤45 days after return to the United States, seven of these patients reported no history of previous illness, suggesting an acute infection and possible primary prophylaxis failure; two of seven patients did not adhere to the chemoprophylaxis regimen, and two patients did not provide adherence information. Of the seven *P. vivax* or *P. ovale* patients with acute onset ≤45 days after return and no history of malaria who took some chemoprophylaxis, three took mefloquine, two took doxycycline, and two took atovaquone-proguanil. Of three patients who reported complete adherence to a correct chemoprophylaxis regimen, one patient traveled for tourism to India for an unknown period and took atovaquone-proguanil and two patients traveled to West Africa for ≤2 months, one of whom took mefloquine and did not report the reason for travel and one of whom took doxycycline and traveled to visit friends and relatives while on leave from the U.S. military. Resistance markers for *P. vivax* and *P. ovale* infections are not well defined, and no antimalarial resistance testing was performed on samples from these three patients. Possible explanations for acute *Plasmodium* infections in patients who adhered to chemoprophylaxis include inadequate dosing or malabsorption of the chemoprophylaxis medication, inaccurate reporting of adherence, emerging parasite resistance, or chemoprophylaxis failure.

#### *P. falciparum, P. malariae,* or Mixed Infections After CDC-Recommended Prophylaxis Use

Among the 162 patients with identified *Plasmodium* species who reported taking a CDC-recommended prophylaxis regimen, 122 (75.3%) of the infections were from *P. falciparum*, and 10 (6.2%) were from *P. malariae.* Among the patients with *P. falciparum* infections, 120 (98.4%) reported travel to Africa, and 74 (60.7%) of these had traveled to West Africa; two *P. falciparum* cases occurred after travel to the Caribbean (one each to Haiti and the Dominican Republic). Of 108 *P. falciparum* case reports containing information on adherence to the recommended prophylaxis regimen, 66 (61.1%) patients were not adherent. A total of 42 cases of *P. falciparum* occurred among patients who reported complete adherence to a recommended chemoprophylaxis regimen; 16 (38.1%) patients took mefloquine, 15 (35.7%) took doxycycline, and 11 (26.2%) took atovaquone-proguanil. All 42 patients with *P. falciparum* infections who adhered to chemoprophylaxis acquired their infections from Africa, with 26 (61.9%) from the West Africa region. Of the 16 infections acquired by patients who adhered to mefloquine, nine were from West Africa, three were from Kenya, two were from Cameroon, and one each was from Equatorial Guinea and Uganda. Of the 15 infections acquired among patients who adhered to the doxycycline regimen, nine were from West Africa. Of the 11 patients with malaria who adhered to atovaquone-proguanil for chemoprophylaxis, eight cases were acquired from West Africa, and one case each was acquired from Cameroon, Uganda, and Africa, country unspecified. CDC received three specimens for molecular resistance surveillance from patients with these infections who adhered to chemoprophylaxis; two patients took mefloquine and one took doxycycline for chemoprophylaxis. Of the two who reported adherence to mefloquine for prophylaxis, both had been to Kenya for short-term travel (i.e., ≤3 weeks); one person traveled for tourism, and one did not specify a reason for travel. Of two samples tested, one had a *pfmdr1* copy number amplification, which is known to be associated with mefloquine resistance. Valid genetic markers are not available to assess one specimen from a person who took doxycycline for chemoprophylaxis. No specimens from the patients who adhered to atovaquone-proguanil were provided for molecular resistance testing.

A total of 10 cases of *P. malariae* occurred after patients had taken a recommended prophylaxis regimen. All 10 patients had information on adherence; five patients reported taking all doses of chemoprophylaxis, and five did not adhere to the regimen. Of the five patients who were adherent to chemoprophylaxis, two took atovaquone-proguanil, two took mefloquine, and one took doxycycline. All five had traveled to African countries outside of West Africa (one case each from Cameroon, Malawi, Sudan, Tanzania, and Africa, country unspecified). Specimens were not tested at CDC for any of the patients who reported adherence. Possible explanations for *Plasmodium* infections in patients who adhered to chemoprophylaxis include inadequate dosing or malabsorption of the chemoprophylaxis medication, inaccurate reporting of adherence, emerging parasite resistance, or chemoprophylaxis failure.

### Patients with a Recent History of Malaria

Among the 2,112 cases of malaria imported into the United States in 2017, reports for 1,488 (70.5%) provided information on previous history of malaria; 302 (20.3%) reported having had malaria during the past 12 months. The infecting species for the previous illness was reported for 45 (14.9%) of 302 patients with a previous history of malaria; 24 (53.3%) recalled *P. falciparum*, 15 (33.3%) recalled *P. vivax,* five (11.1%) recalled *P. malariae,* and one (2.2%) recalled *P. ovale*. Of the 16 patients who recalled having a previous *P. vivax* or *P. ovale* infection, all were currently infected with the same species and were likely relapses. Among the 16 likely *P. vivax* or *P. ovale* relapses, six patients reported complete dates for the current and former illness onsets, and the mean number of days between the previous and relapsing illness was 135 days (minimum of 54 and maximum of 258 days). Of 16 patients who recalled having a previous *P. vivax* or *P. ovale* infection, 15 provided information on the country of acquisition for the current infection; eight infections were acquired from Asia, four from South America, two from Africa, and one from Oceania. Of 16 patients with *P. vivax* or *P. ovale* infections, five had documentation of receiving primaquine for the current illness to prevent relapse. In one case, an immigrant arriving in the United States from India in 2016 had three *P. vivax* illnesses in 2017, occurring in February, April, and August. The April and August illnesses were classified as relapses. Treatment medications were not reported for this patient’s first two illnesses; however, primaquine, to prevent relapses, was documented for treatment of the August (third) illness, and the patient recovered.

Among the 24 patients who recalled a *P. falciparum* infection during the past 12 months, 16 (66.7%) had a current infection with *P. falciparum,* four (16.7%) had *P. ovale*, two (8.3%) had *P. vivax*, and one (4.2%) each had *P. malariae* or an unknown species reported for the current infection. Among the five patients who recalled a previous *P. malariae* infection during the past 12 months, four (80.0%) were currently infected with *P. malariae,* and one (20.0%) was infected with *P. vivax*. The species recall for the previous infections were not validated; therefore, a larger number of patients might have experienced a relapse, especially those who were currently infected with *P. ovale* or *P. vivax* (four and three patients, respectively). Of 29 persons who recalled having a previous infection with *P. falciparum* or *P. malariae*, 28 had information on the country of acquisition for the current infection; 23 (82.1%) acquired their current infection from Africa, three (10.7%) from Asia, and two (7.1%) from South America. Persons who were infected with nonrelapsing species (*P. falciparum* and *P. malariae*) could have been infected again during the past 12 months while they were in a country where malaria is endemic. Of these 29 patients, four had illnesses reported in the United States earlier in 2017 or in 2016; the other patients might have had earlier illnesses while outside of the United States, or, if a patient experienced an earlier malaria illness within the United States, then the available information might not have been sufficient to match the patient to the same person.

### Reason for Travel

Among nonmilitary U.S. residents (i.e., civilians), the reason for travel to a country where malaria is endemic was reported for 1,038 (81.0%) of 1,282 imported malaria cases (Table 6). Of these U.S. civilians with known reason for travel , VFR was reported as the reason for travel for 758 (73.0%), missionary travel was reported for 91 (8.8%), business travel was reported for 88 (8.5%), and tourism was reported for 60 (5.8%) patients. A total of 27 (2.6%) patients acquired malaria during travel for education (as students or teachers), three (0.3%) cases were related to Peace Corps service in 2017, and one (<1.0%) case occurred among airline or ship crew members. A total of 10 patients provided other reasons for travel (1.0%); nine of these reasons included humanitarian or volunteer work, and one was to seek medical treatment. Of the 758 malaria patients who reported VFR travel, 709 (93.5%) had traveled to Africa, 31 (4.1%) to Asia, and approximately 1% or less had traveled to Central America and the Caribbean, South America, or Oceania. Of malaria patients who traveled for missionary reasons or as part of a church group, 86% went to Africa. In 2017, of the 60 malaria patients who traveled for tourism, 44 (73.3%) had traveled to Africa, seven (11.7%) to South America, five (8.3%) to Central America and the Caribbean, and two (3.3%) to Asia.

**TABLE 6:** Number* and percentage of imported malaria cases among U.S. civilians and non-U.S. residents, by purpose of travel at the time of acquisition — United States, 2017

Category	U.S. civilians	Non-U.S. residents
	No. (%)	No. (%)
Visiting friends and relatives	758 (59.1)	144 (28.0)
Tourist	60 (4.7)	16 (3.1)
Missionary or dependent	91 (7.1)	6 (1.2)
Business	88 (6.9)	18 (3.5)
Student or teacher	27 (2.1)	33 (6.4)
Air crew or sailor	1 (0.1)	3 (0.6)
Peace Corps	3 (0.2)	0 (0)
Refugee or immigrant	0 (0)	186 (36.1)
Military (non-U.S.)^†^	NA	2 (0.4)
Other	10 (0.8)	1 (1.4)
Unknown	244 (19.0)	106 (20.6)
**Total**	**1,282 (100)**	**515 (100)**

**Abbreviation:** NA = not applicable.

* N = 1,797.

^†^ Foreign residents who traveled to the United States for military reasons.

Among the 515 imported cases among non-U.S. residents, reason for travel was available for 409 (79.4%) cases (Table 6). Among those non-U.S. residents who provided a reason for travel, traveling to the United States as an immigrant or a refugee was cited in 186 case reports (45.5%), a decrease from 2016 (263 [55.5%]). Traveling from countries where malaria is endemic to the United States to visit friends and relatives was reported by 144 (35.2%) patients, an increase from 2016 (118 [ 24.9%]). A total of 33 (8.1%) patients traveled for education (as a student or teacher) in 2017. Tourism was reported by 16 (3.9%) patients, missionary or travel with a church group was reported by six (1.5%) patients, and airline or ship crew travel was reported by three (<1.0%) patients. Another reason for travel was reported by one (<1.0%) patient. Of the 186 non-U.S. residents who traveled to the United States as a refugee or an immigrant, 140 (75.3%) originated in Africa, followed by Asia (35 [18.8%]) and the Americas and Caribbean (nine [4.8%]); two persons who traveled as refugees or immigrants did not report a country of origin.

### Malaria by Age, Ethnicity, and Race

Age was reported for all 2,161 (100%) of the patients with malaria in 2017, and of these, adults aged ≥18 years accounted for 1,781 (82.4%) cases. Among adult malaria cases, one was transfusion induced and two cases were cryptic. A total of 67 (3.1%) patients were aged <5 years, and 146 (6.8%) were aged ≥65 years. Of the 380 (17.6%) children (aged <18 years), 150 (39.5%) were U.S. residents, and 134 (89.3%) of these had traveled to Africa. Among the U.S. resident children, 146 cases were imported, two were congenital, one was transfusion induced, and one was cryptic. Of the 146 imported cases among U.S. resident children aged <18 years, 102 (69.9%) were VFR travelers, nine (6.2%) traveled for education, five (3.4%) traveled for tourism, and three (2.1%) traveled as a dependent for missionary or religious purposes. Travel for volunteer humanitarian reasons was reported for one child, and no reason was provided for 26 children. Among these 146 children, information on chemoprophylaxis use was provided for 134 (89.3%); 82 (61.2%) did not take medication during travel to prevent malaria. Of the 52 (38.8%) children who took a chemoprophylaxis regimen, 31 (59.6%) took a CDC-recommended antimalarial that was appropriate for the region of travel, 19 (36.5%) did not report the regimen taken, and two took an incorrect medication for the country of travel. Of 98 children who were VFR travelers, 40 (40.8%) took any chemoprophylaxis medication, whereas 12 (33.3%) of 36 non-VFR travelers aged <18 years took any chemoprophylaxis. Of the 28 children with information on adherence, case reports from 12 (42.9%) indicated that all doses were taken; nine (75.0%) of 12 children who adhered were VFR travelers. Of 12 cases in children who adhered to prophylaxis, 11 cases were acquired from Africa, and one was acquired from Asia; nine of these cases were confirmed *P. falciparum*, and one each were *P. vivax*, *P. ovale*, and *P. malariae*. Among children, three cases were severe, and no deaths were reported.

An asymptomatic child aged 7 years who immigrated to the United States from the Democratic Republic of Congo was tested as part of an adoption screening process and was infected with multiple species. The CDC reference laboratory confirmed infection with *P. falciparum, P. ovale,* and *P. malariae* species by PCR. The child was treated with atovaquone-proguanil.

Hispanic or Latino ethnicity information was provided for 1,793 (83.0%) of 2,161 cases. A total of 35 persons reported Hispanic or Latino ethnicity; 19 (54.3%) infections were acquired from Africa, seven (20.0%) from Central America or the Caribbean, six (17.1%) from South America, one (2.9%) from Asia, and two (5.7%) from an unknown region. Of the patients reported as having Hispanic or Latino ethnicity, 22 were U.S. residents, 12 were non-U.S. residents, and one did not have residence status reported.

Race information was available for 1,884 (87.2%) cases; of these, 1,480 (78.6%) cases were Black or African American, 265 (14.1%) were White, 129 (6.9%) were Asian, and <1.0% were American Indian/Alaskan Native or Hawaiian/Pacific Islander. Of the 1,290 U.S. residents, information on race was available for 1,173 (90.9%). Of these, 910 (77.6%) were Black or African American, 213 (18.2%) were White, 45 were Asian, and <1.0% were other races. Of the 707 U.S. residents with race information and who had traveled to visit friends and relatives, 666 (94.2%) were Black or African American, 21 (2.8%) were White, and 19 (2.7%) were Asian; <1.0% were other races.

Among all cases with race information in 2017, of the 1,480 Black or African American patients, 1,403 (94.8%) had traveled to Africa. Of the 129 cases among Asian patients, 106 (82.2%) had traveled to Asia, and 17 (13.2%) had traveled to Africa. Persons who were reported as White (265 cases) predominantly traveled to Africa (214 [80.8%]); 5.3% of White patients had traveled to South America (14 cases), 4.9% to Asia (13 cases), and 3.8% to Central America and the Caribbean (10 cases).

### Hospitalization

In 2017, hospitalization information was reported for 1,904 (88.1%) of 2,161 confirmed cases; of these, 1,302 (68.4%) patients were hospitalized for their malaria illness. A total of 972 (74.7%) hospitalized patients had *P. falciparum* malaria in 2017, and 278 (21.4%) hospitalized patients had one or more signs or symptoms of severe malaria. No significant change in these percentages occurred from 2016 to 2017; in 2016, a total of 1,261 [68.9%] patients were hospitalized among 1,831 with this information reported, 918 (72.8%) with *P. falciparum* and 268 (21.3%) with severe malaria (not mutually exclusive).

### Treatment for Uncomplicated Malaria Cases

#### Overall

Among the 2,161 confirmed malaria cases in 2017, a total of 1,849 (85.6%) cases were uncomplicated. Of these, 1,567 (84.8%) had antimalarial treatment information indicated on the case report form. The most common treatment administered for uncomplicated malaria in 2017 was atovaquone-proguanil, used to treat 966 (61.7%) patients with treatment information, an increase from 2016 when 879 (58.1%) atovaquone-proguanil treatments were reported. Quinine-based treatment was the second most common; 237 (15.1%) patients were treated with this regimen. Artemether-lumefantrine, the only artemisinin-based combination therapy approved by FDA for treatment of uncomplicated malaria in the United States, was used for 200 (12.8%) patients. A total of 111 (7.1%) uncomplicated cases were treated with chloroquine, and 82 (5.2%) were treated with mefloquine.

The *CDC Guidelines for Treatment of Malaria in the United States*, herein referred to as the CDC guidelines, provides guidance for the treatment of malaria according to species, disease severity, pregnancy status, and region of acquisition (*31*). Of the 1,567 uncomplicated cases with treatment information, 1,398 (89.2%) were treated in accordance with CDC guidelines, a similar proportion to the number of uncomplicated cases treated correctly in 2016 (1,365 [90.2%]). Of those treated correctly in 2017, a total of 183 (13.1%) patients reported taking an additional antimalarial medication that exceeded what is recommended by the CDC guidelines. Among the 169 (10.8%) patients whose treatment was not in accordance with CDC recommendations, six (3.6%) were treated with the same antimalarial that the patient had used for chemoprophylaxis. To avoid potential toxicity and reduced efficacy, patients should not be treated with the same antimalarial that was used for chemoprophylaxis (*31*).

#### Adequacy of Treatment by Species

Among the 1,072 uncomplicated *P. falciparum* cases, 972 (90.7%) were treated according to CDC guidelines, and 125 (12.9%) of these patients received an antimalarial treatment that exceeded the recommendations. Of 100 *P. falciparum* patients who were not treated in accordance with CDC recommendations, three (3.0%) were pregnant. Treatment followed CDC guidelines for 36 (92.3%) of 39 cases of uncomplicated *P. malariae* infections*;* nine (25.0%) of those patients received an additional antimalarial medication. Of those with *P. ovale* and *P. vivax* infections, 91 (89.2%) and 153 (86.9%), respectively, were treated in accordance with CDC guidelines for their acute infection; one (1.1%) patient with a *P. ovale* infection and 12 (7.8%) patients with *P. vivax* infections received an additional acute-phase antimalarial medication. Administration of primaquine to treat relapsing illness (the liver hypnozoite stage of the parasite) was reported for 68 (39.3%) of all 173 nonpregnant patients with *P. vivax* infections and treatment information. Among the 101 nonpregnant patients with *P. ovale* infections, primaquine was administered to 23 (22.8%). Of 16 mixed infections with recorded treatment information, 13 (81.3%) were treated according to CDC guidelines; seven (53.9%) of these patients received an additional antimalarial to treat the acute illness. Malaria infections with an unknown species should be treated following recommendations for *P. falciparum* (*31*); 133 (82.1%) of 162 cases with unknown infecting species were treated according to recommendations, and 29 (21.8%) of these were treated with an additional antimalarial for the acute illness. Of 159 nonpregnant patients with unknown infecting species, 20 (12.6%) were treated with primaquine.

### Severe Malaria

In 2017, a total of 312 (14.4%) of 2,161 cases were classified as severe malaria. Of the patients with severe malaria, six (1.9%) died. One patient who died, for a total of seven deaths, was considered to have had uncomplicated malaria and died from other causes. Among severe cases, 242 (77.6%) occurred in adults aged 18–64 years. However, children <18 years were more likely to have severe malaria than adults (70 of 380 [18.4%] children aged <18 years versus 242 of 1,781 [13.6%] adults aged ≥18 years). The proportion of adults aged ≥65 years with severe malaria was 16.4% (24 of 146 cases); the proportion of severe malaria among patients aged <65 years was 14.3% (288 of 2,015 cases). In 2017, a similar proportion of patients with severe malaria were U.S residents or non-U.S. residents; 208 (15.8%) of 1,316 U.S. residents, compared with 67 (13.0%) of 516 non-U.S. residents, had severe malaria. Among patients with severe malaria in 2017, a total of 285 (91.4%) had *P. falciparum* infections, 12 (3.9%) had *P. vivax* infections, and 10 (3.2%) were infected with an unknown *Plasmodium* species. Less than 1.0% of severe infections were caused by *P. malariae* (n = 2), *P. ovale* (n = 2), or mixed species (n = 1). In 2017, the species distribution among severe cases was similar to the distribution in 2016. All six patients who died of severe malaria in 2017 had *P. falciparum* infections.

Among 207 U.S. residents with imported severe malaria and information on chemoprophylaxis (66.3% of 312 severe cases), 44 (18.6%) reported having taken any medication for chemoprophylaxis; 18 (40.9%) of these did not provide adherence or chemoprophylaxis medication information. Among 26 patients with severe malaria and known chemoprophylaxis adherence information, 69.2% (18 cases) did not adhere to or take a recommended regimen for malaria prevention, compared with 60.4% (90 of 149) of persons (U.S. residents and imported cases) with uncomplicated malaria who did not adhere to chemoprophylaxis or take the correct regimen. Of the eight patients with severe malaria who adhered to a recommended chemoprophylaxis regimen, three each took atovaquone-proguanil or doxycycline, and two patients took mefloquine. One specimen from a patient who took doxycycline was tested at CDC, although no validated markers for doxycycline resistance are known. Potential reasons for chemoprophylaxis failure in these patients with severe malaria include inadequate dosing or malabsorption of the chemoprophylaxis medication, inaccurate reporting of adherence, or emerging parasite resistance.

Patients with severe malaria can have multiple clinical complications; among reported patients with severe malaria in 2017, acute kidney injury was most common and was experienced by 47 (15.1%) patients. Cerebral malaria was reported for 33 (10.6%) patients, acute respiratory distress syndrome for 25 (8.0%), severe anemia for 13 (4.2%), and jaundice for 12 (3.9%). Case reports from 256 patients with severe malaria contained information on the percentage of *Plasmodium* species parasitemia, of which 176 (68.8%) had ≥5% parasitized red blood cells. CDC guidelines in 2017 stated that patients with severe malaria should be treated aggressively in an inpatient setting with intravenous quinidine gluconate or artesunate. In 2017, a total of 24 (7.7%) patients with severe malaria were not hospitalized; the hospitalization status for another 10 (3.2%) patients was unknown. At that time, the only FDA-approved medication for this indication was intravenous quinidine gluconate; for patients who could not tolerate quinidine gluconate or when quinidine was not available, artesunate could be obtained from CDC through an investigational new drug protocol (*57*). In 2017, a total of 165 (54.1%) patients with severe malaria were treated with quinidine gluconate, 21 (6.9%) were treated with artesunate, and 24 (7.9%) were treated with both quinidine gluconate and artesunate. A total of 210 (67.3%) patients with severe malaria were treated with a parenteral antimalarial regimen, and 95 (30.4%) were treated with an oral regimen alone; treatment information was unknown for seven (2.2%) patients. Among six patients with severe malaria who died, three were treated with quinidine, one with artesunate, and two with both quinidine and artesunate. Among 27 pregnant women, four had severe malaria, of whom two were treated with quinidine and two with quinidine and artesunate.

In 2017, a total of 168 (55.1%) of 305 patients with severe malaria and information on treatments received appropriate treatment, a comparable proportion to 2016, when 147 (49.7%) of 296 patients received appropriate treatment. In 2017, patients with severe malaria were significantly more likely to have inappropriate treatment (137 [44.9%] of 305) than patients with uncomplicated malaria (167 [10.7%] of 1,565). Although all malaria *Plasmodium* species can cause severe malaria, the rapid proliferation of *P. falciparum* parasites requires timely diagnosis and treatment to prevent progression to hyperparasitemia and severe illness. In 2017, on average, inpatients with *P. falciparum* infections were hospitalized 5.2 days after illness onset, regardless of disease severity (with a mean of 5.1 days and median of 3 days for uncomplicated cases, and a mean of 5.4 days and median of 4 days for severe cases).

Among the 312 patients with severe illness, 293 (93.9%) infections were acquired from Africa; 11 (3.5%) from Asia; and <1.0% from the Caribbean (three cases), South America (two cases), and Central America (one case). Infection from an unknown region was acquired by two patients with severe malaria. Among the 258 (82.7%) patients with severe malaria who reported a reason for travel, 142 (55.0%) reported VFR as the primary reason for travel, of whom 134 (94.4%) had acquired the infections from Africa, and 99 (69.7%) of these were from West Africa. A total of 26 (10.1%) patients with severe illness traveled for business, and 23 (8.9%) each traveled for tourism or for missionary or religious purposes. Of patients with severe malaria, 22 (8.5%) traveled to the United States as immigrants or refugees, and 15 patients (5.8%) traveled for education (as a student or teacher). Less than 1.0% of patients with severe cases traveled as members of the U.S. military (three cases), as crew of an airline or ship (one case), or for another reason (three cases).

### Malaria During Pregnancy

Among 805 women with malaria diagnosed in the United States in 2017, a total of 27 (3.4%) were pregnant, a decrease from 2016 when 50 (6.3%) women with malaria were pregnant. In 2017, a total of 22 (81.5%) pregnant women were hospitalized, including all four (14.8%) with severe malaria; all pregnant women with malaria recovered. Data on birth outcomes are not systematically collected.

Among pregnant women, 20 (74.1%) had *P. falciparum* infections, three (11.1%) were infected with *P. vivax*, one (3.7%) with *P. ovale,* and three with an unspecified *Plasmodium* species. Approximately half of the pregnant women (14 [51.9%]) were non-U.S. residents, and 10 (37.0%) were U.S. residents; residency status was not provided for three (11.1%) women. Of 20 (74.1%) pregnant women who indicated a reason for travel, 10 (50.0%) were refugees or immigrants, nine (45.0%) traveled to visit friends and relatives, and one (5.0%) traveled for business.

Due to the safety profile of medications approved for pregnancy and antimalarial resistance patterns, antimalarial drug choices to prevent or treat malaria during pregnancy are limited. In most areas where malaria is endemic, only mefloquine is approved for chemoprophylaxis; in 2017, either mefloquine or quinine with clindamycin was recommended as treatment for uncomplicated malaria during pregnancy (*31*). Chloroquine for chemoprophylaxis or treatment of uncomplicated malaria is only recommended for pregnant women to use when travel is within areas with chloroquine-sensitive malaria. Primaquine can cause hemolytic anemia among persons with glucose-6-phosphate dehydrogenase (G6PD) deficiency and should not be administered during pregnancy. Severe malaria in pregnant women should be treated aggressively with intravenous regimens; in the United States during 2017, the options were parenteral quinidine-gluconate or artesunate. Of 10 pregnant women who were U.S. residents, nine (90.0%) provided information on chemoprophylaxis use; eight women did not take chemoprophylaxis to prevent malaria during travel, and one patient reported adhering to an unnamed medication for prevention of malaria.

Treatment information was available for all 27 pregnant women with malaria. Of the 23 patients with uncomplicated malaria, 17 (73.9%) were treated in accordance with CDC guidelines, which is a similar proportion to nonpregnant women (75.9%) who were treated according to guidelines. Among the six pregnant women whose treatment was not in accordance with CDC guidelines, two patients received atovaquone-proguanil, which is not recommended for use during pregnancy; one woman was treated with a chloroquine-based regimen, which was not appropriate for the region of travel; and one woman with uncomplicated malaria was treated with artemether-lumefantrine, which at the time of treatment in 2017 was not recommended for use in pregnant women but subsequently became endorsed for use in pregnant women in 2018 (*58*). Two pregnant women were treated with primaquine for uncomplicated *P. falciparum* and an unknown *Plasmodium* species infection; primaquine is contraindicated for pregnancy and should be administered after species confirmation of *P. vivax* or *P. ovale* infections.

All four pregnant women with severe malaria were infected with *P. falciparum* and were treated with a parenteral antimalarial; two were treated with quinidine and two with quinidine and artesunate. In addition to the parenteral antimalarial, one woman with severe malaria was treated with atovaquone-proguanil, which is not recommended for treatment during pregnancy.

### Drug Resistance Markers

CDC received 143 (6.6%) whole blood samples for analysis of molecular markers of resistance from patients who received a malaria diagnosis in 2017. Of these, 19 samples were from patients who did not have *P. falciparum* infections, and samples from three patients did not have high enough parasite density to amplify markers of resistance. Molecular resistance testing was performed on 117 *P. falciparum*-only samples and four mixed-infection samples. However, not all loci amplified for each of the markers analyzed. The region of acquisition was known for 114 (97.4%) of 117 specimens tested for antimalarial resistance (Table 7). Of the 111 samples that amplified for pyrimethamine resistance, 108 (97.3%) contained polymorphisms associated with pyrimethamine resistance, and 105 (94.6%) of these had three or more pyrimethamine resistance markers. A total of 111 samples amplified for sulfadoxine resistance analysis, and 77 (69.4%) had at least one marker associated with sulfadoxine resistance; 15 (13.5%) of these had three or more sulfadoxine resistance markers. Of 114 samples tested for chloroquine resistance markers, 38 (33.3%) had a resistant genotype. Copy number variation of the mefloquine locus was assessed for 112 samples, and three (2.7%) contained the resistant genotype. The genes associated with atovaquone resistance were amplified for 108 samples, and three (2.8%) samples had polymorphisms associated with resistance. None of the 114 samples investigated for artemisinin resistance had *pfk13* mutations associated with resistance (Table 7).

**TABLE 7:** Antimalarial drug resistance marker results among *Plasmodium falciparum* specimens, by drug and region of malaria acquisition* — United States, 2017

Resistance markers	Region						
	Africa	Asia	Central American and the Caribbean	South America	Oceania	Unknown	Total
	No. (%)	No. (%)	No. (%)	No. (%)	No. (%)	No. (%)	No. (%)
**Pyrimethamine (n = 111)**	**107 (96.4)**	**1 (0.9)**	**1 (0.9)**	**0 (0)**	**0 (0)**	**0 (0)**	**111 (100)**
No resistance markers	3 (2.7)	0 (0)	0 (0)	0 (0)	0 (0)	0 (0)	**3 (2.7)**
1 resistance marker	1 (0.9)	0 (0)	0 (0)	0 (0)	0 (0)	0 (0)	**1 (0.9)**
2 resistance markers	1 (0.9)	1 (0.9)	0 (0)	0 (0)	0 (0)	0 (0)	**2 (1.8)**
3 or more resistance markers	102 (91.9)	0 (0)	1 (0.9)	0 (0)	0 (0)	2 (1.8)	**105 (94.6)**
**Sulfadoxine (n = 111)**	**106 (95.5)**	**2 (1.8)**	**1 (0.9)**	**0 (0)**	**0 (0)**	**2 (1.8)**	**111 (100)**
No resistance markers	32 (28.8)	0 (0)	1 (0.9)	0 (0)	0 (0)	1 (0.9)	**34 (30.6)**
1 resistance marker	41 (36.9)	2 (1.8)	0 (0)	0 (0)	0 (0)	0 (0)	**43 (38.7)**
2 resistance markers	18 (16.2)	0 (0)	0 (0)	0 (0)	0 (0)	1 (0.9)	**19 (17.1)**
3 or more resistance markers	15 (13.5)	0 (0)	0 (0)	0 (0)	0 (0)	0 (0)	**15 (13.5)**
**Chloroquine (n = 114)**	**108 (94.7)**	**2 (1.8)**	**1 (0.9)**	**0 (0)**	**0 (0)**	**1 (0.9)**	**114 (100)**
No resistance markers	73 (64.0)	1 (0.9)	1 (0.9)	0 (0)	0 (0)	1 (0.9)	**76 (66.7)**
1 or 2 resistance markers	0 (0)	1 (0.9)	0 (0)	0 (0)	0 (0)	0 (0)	**1 (0.9)**
3 resistance markers	35 (30.7)	0 (0)	0 (0)	0 (0)	0 (0)	2 (1.8)	**37 (32.5)**
**Mefloquine (n = 112)**	**107 (95.5)**	**2 (1.8)**	**1 (0.9)**	**0 (0)**	**0 (0)**	**2 (1.8)**	**112 (100)**
No resistance markers	104 (92.9)	2 (1.8)	1 (0.9)	0 (0)	0 (0)	2 (1.8)	**109 (97.3)**
1 resistance marker	3 (2.7)	0 (0)	0 (0)	0 (0)	0 (0)	0 (0)	**3 (2.7)**
**Atovaquone (n = 108)**	**103 (95.4)**	**2 (1.9)**	**1 (0.9)**	**0 (0)**	**0 (0)**	**2 (1.9)**	**108 (100)**
No resistance markers	101 (93.5)	2 (1.9)	1 (0.9)	0 (0)	0 (0)	1 (0.9)	**105 (97.2)**
1 resistance marker	2 (1.9)	0 (0)	0 (0)	0 (0)	0 (0)	1 (0.9)	**3 (2.8)**
**Artemisinin (n = 114)**	**107 (93.9)**	**3 (2.6)**	**1 (0.9)**	**0 (0)**	**0 (0)**	**3 (2.6)**	**114 (100)**
No resistance markers	107 (93.9)	3 (2.6)	1 (0.9)	0 (0)	0 (0)	3 (2.6)	**114 (100)**

* The region of acquisition was known for 114 (97.4%) of 117 specimens tested for antimalarial resistance.

Because of widespread resistance to pyrimethamine and sulfadoxine, CDC does not recommend using drugs containing these components to treat malaria in the United States (*31*). None of the patients with chloroquine resistance markers had exposure to malaria in a chloroquine-sensitive region. Of the samples without resistance markers to chloroquine, 73 (96.1%) were from patients who acquired malaria in Africa, and one (1.3%) each was from Asia, Central America, and an unknown region. The three patients with markers of mefloquine resistance (>1 copy of the *pfmdr1* gene) had traveled to Kenya, Ghana, and Nigeria. Of these three, one reported taking mefloquine for chemoprophylaxis and reported complete adherence, suggesting that the illness could have been sustained because of the parasites’ resistance to mefloquine. No patients with the mefloquine-resistant genotype were treated with mefloquine, and all recovered from their illness. Information on travel, chemoprophylaxis, and treatment medications was available for two of the three patients who acquired malaria with genetic markers for atovaquone resistance. They had traveled to Ghana and Guinea and did not report having taken chemoprophylaxis. Of these two patients, one was treated with artemether-lumefantrine and one with atovaquone-proguanil. For the patient who had traveled to Guinea and was treated with atovaquone-proguanil, no treatment failure was indicated.

### Selected Malaria Case Reports

#### Congenital Cases

In 2017, two cases of congenital malaria were reported. Parasites were transmitted from mother to child during pregnancy or during labor and delivery.

**Case 1.** A pregnant woman aged 23 years immigrated to the United States from Pakistan in late 2016. Approximately 7 months later she became ill, and 4 days after symptom onset she gave birth and received a diagnosis by blood smear and PCR of *P. vivax* malaria with 4% parasitemia. The woman was treated with atovaquone-proguanil oral (per os [PO]); however, because clinicians believed atovaquone-proguanil prompted her to have hypoglycemia, her treatment was changed to quinine PO and doxycycline PO. Primaquine PO was administered, and the patient was advised to pump and dispose of her breast milk for 18 days. One day after birth, a full-term female infant that weighed 5 lb, 12 oz, at birth was tested for malaria using a blood smear, and no parasites were detected. Approximately 4 weeks after birth, the infant experienced fever and had symptoms of anemia and thrombocytopenia. Blood smear showed *P. vivax* with 5% parasitemia. The infant was admitted to the perinatal intensive care unit and administered hydroxychloroquine PO via feeding tube, intravenous (IV) clindamycin, and IV quinidine and monitored for cardiac arrythmias. After completion of quinidine, the infant was transferred from the perinatal intensive care unit to the pediatric inpatient ward, where she completed a 7-day course of IV clindamycin without cardiac monitoring. Repeat laboratory tests showed resolution of parasitemia and improvement in anemia and thrombocytopenia, and the infant was discharged after 8 days in the hospital.

**Case 2.** An Asian male infant aged 18 days, born full-term, whose mother was asymptomatic and had immigrated to the United States from Pakistan 1 year earlier, developed a rash on his abdomen and chin. His primary care physician diagnosed staph pustulosis and discharged him. That night he developed a fever of 103°F (39.4°C) and did not wake to eat. The next morning, he was returned to the primary care physician, where he was febrile and referred to the emergency department. Laboratory results showed elevated liver enzymes (aspartate aminotransferase and alanine aminotransferase) and blood urea nitrogen, and thrombocytopenia. Blood smear identified *P. vivax* parasites with <5% parasitemia. The infant was administered chloroquine PO in the hospital and IV antibiotics, including clindamycin, for the staph skin infection. The mother was counseled to obtain treatment for malaria with chloroquine and primaquine, although she has no record of a malaria diagnosis. The infant was discharged from the hospital after 4 days.

#### Transfusion-Transmitted Malaria

In 2017, two cases of TTM occurred through blood transfusions in the United States.

**Recipient 1.** A report of this case and investigation was published (*10*). A Black male aged 15 years with erythrocytapheresis-dependent sickle cell disease sought care in the emergency department for chest pain and malaise. He had no history of travel but had received monthly red blood cell transfusions, most recently, 24 days previously. He was discharged after an evaluation for acute chest syndrome but did not have the condition. Four days later, he was reevaluated for fever (100°F [37.8°C]) and back pain. At that time, during routine review of preerythrocytapheresis blood samples, ring trophozoite parasites were observed. Blood smear microscopy identified *Plasmodium* parasites with 0.5% parasitemia. Whole blood samples were sent to the state public health laboratory and were confirmed to be positive for *P. falciparum* and negative for *Babesia microti.* The patient did not have symptoms of severe malaria; he was treated with atovaquone-proguanil PO and recovered.

**Investigation 1.** The blood center performed a traceback investigation of the donors and identified eight donors who provided red blood products to the recipient, all of whom were deferred from further donations pending the investigation. Five of eight collection tube segments, remaining from the initial blood product (which underwent dilution and processing), were available for testing. All eight donors were contacted to request a follow-up blood sample, of whom five provided a sample months after the initial donation. Altogether, seven of eight donors had specimens that were evaluated. Of the six donors’ specimens tested by immunofluorescence assay (IFA) for the presence of malaria antibodies, five were negative for all *Plasmodium* species tested. Five of six donors’ specimens tested by PCR (nested and real-time PCR [RT-PCR]) had only negative results. The sole donor who had specimens test positive (by IFA and PCR) had a red blood cell segment sample and a fresh blood sample available for testing. The segment sample was positive by IFA for *P. falciparum* at a titer of 1:64 and had an RT-PCR cycle threshold value of 40.9 for *P. falciparum*, which is at the limit of detection. The follow-up whole blood sample was positive for *P. falciparum* by IFA at a dilution titer of 1:1,024. PCR was conducted four times on the whole blood sample from this donor (RT-PCR three times and nested PCR one time), and *P. falciparum* parasites were detected in one of four PCR tests. Because the donor specimen had low parasite density, performing extensive molecular genotyping to match the parasites between the recipient and donor was not possible. However, genetic marker analysis of *merozoite surface protein 1* and *merozoite surface protein* *2* showed loci amplicon sizes of approximately 180 and 300 base pairs, respectively, for both the recipient and donor specimens.

**Donor 1.** The implicated donor in this case of TTM was an asymptomatic, first-time donor, born in West Africa. The donor questionnaire, which was completed in early 2017 at the time of the blood donation, showed that the donor denied being outside of the United States or Canada during the previous 3 years or ever having malaria. During the investigation, another interview determined that the donor had immigrated from West Africa in mid-2014 and had experienced previous episodes of malaria at unknown times. The donor was treated with atovaquone-proguanil PO for uncomplicated malaria.

**Recipient 2.** At the end of 2016, a White male child aged 14 months was admitted for respiratory failure that required extracorporeal membrane oxygenation (ECMO) for 22 days in early 2017 and transfusion with 48 units of packed red blood cells. Approximately 1 month after the start of ECMO, the patient developed fever and was treated with multiple antibiotics, and while the blood cultures remained negative, fevers continued. The patient did not travel outside of his home state. Non-*P. falciparum* parasites were identified on thin and thick blood smears 1 week after fever onset. PCR was used to confirm infection with *P. ovale*. The patient was treated with chloroquine PO and primaquine PO and recovered.

**Investigation 2.** The blood center identified 27 donors who contributed 48 units of packed red blood cells to the recipient in early 2017. All 27 donors were interviewed again by the blood center. Three donors were foreign-born (Iran, India, and Cameroon), and follow-up blood specimens were requested and obtained from the donors from Cameroon and Iran. The donor from Cameroon was considered highest risk because of travel within the last 3 years. IFA and PCR tests were negative for the donor originating from Iran. A specimen from the donor originally from Cameroon tested positive by IFA for *P. falciparum* and *P. ovale* (titers of 1:1,024 and 1:256, respectively). Whole blood from the Cameroonian donor was negative by PCR (two rounds of RT-PCR, one round of nested PCR). No other donor specimens were tested in this investigation.

**Donor 2.** The limited testing performed in this investigation suggests that the recipient’s malaria infection could have originated from the blood donor who had been in Cameroon within the previous 3 years. No additional details about the donor were provided to CDC by the blood center or health department. Whether the implicated donor was referred for antimalarial treatment is unclear.

#### Cryptic Cases

Three cases were classified as cryptic in 2017. The patients had not traveled recently, and investigations revealed a low likelihood of local transmission and no health care or blood exposure (e.g., transfusion, needle stick, intravenous drug use, or home tattooing).

**Case 1.** Twelve days after the onset of unspecified symptoms, a Black male aged 16 years went to the emergency department and was admitted the following day with a febrile illness. The public health laboratory and CDC confirmed that the patient had a *P. falciparum* infection with 2% parasitemia. He was treated with atovaquone-proguanil PO and recovered. The patient was born in the United States, but his parents were from Nigeria and they spent substantial time there. In repeated interviews with health care providers and the local health department, the patient and his guardian reported that the last time he had traveled internationally was to Nigeria, more than 2 years earlier, in 2015, for approximately 15 days; he had never lived in Nigeria or another country where malaria is endemic. The patient stated that he had never had malaria, nor did he have a febrile illness after travel. However, the patient’s father was treated for malaria in Nigeria 1 or 2 years previously and had also received a malaria diagnosis in the United States in 2016, confirmed by the state public health laboratory. Blood specimens for the father (2016 illness) and son (2017 illness) were sent to CDC, and parasite sequencing and microsatellite analysis was conducted. Genotype profiles and microsatellite results showed that the parasites from the father’s 2016 illness and the son’s 2017 illness were not related. The patient traveled to a southern U.S. state 1 month before illness onset, although his infection could not be linked epidemiologically to any malaria case there. The local health department vector control staff conducted mosquito surveillance in the vicinity of the patient’s residence weekly during the month of his 2017 illness and did not find *Anopheles* species mosquitos, which are the type of mosquitos that transmit malaria. Although *Anopheles quadrimaculatus* is present in the region, mosquito surveillance in this county found only two specimens in 2017; both were located outside of city limits and far from the patient’s residence. The health department did not find evidence of local transmission.

**Case 2.** A Black woman aged 23 years had emigrated from the Democratic Republic of Congo to a refugee camp in Tanzania at age 3 years, where she lived until she immigrated to the United States at age 13 years during 2007, which was the last time she reported traveling. Recently, *Schistosoma mansoni* eggs had been identified in her stool, and she was treated with praziquantel; she had splenomegaly, leading to a laparoscopic splenectomy. She sought care at the emergency department 3 days after discharge from the hospital for the splenectomy, which had occurred 1 week previously; she had fever (102.9°F [39.4°C]) and a bifrontal headache that resolved between fevers. The patient was admitted for additional evaluation and management. An infectious disease consult was ordered. Laboratory analysis indicated she was anemic (Hb: 10.6 g/dL, hematocrit: 31.7%) and lymphopenic (3.1% of white blood cells). A chest radiograph and abdominal computerized tomography (CT) scan did not show any abnormalities. She received a malaria diagnosis by blood smear, and PCR at a commercial laboratory confirmed *P. ovale* species; no parasitemia was reported. Primaquine PO was the only antimalarial medication documented in the medical record. Possible explanations for this illness include delayed relapse that opportunistically appeared after splenectomy and nondisclosed travel.

**Case 3**. An Asian man aged 71 years was admitted to the hospital and received a diagnosis by blood smear microscopy and PCR (by the state public health laboratory), confirming *P. malariae* infection with 2% parasitemia. He was treated with chloroquine PO and recovered. The state health department interviewed the patient in Mandarin two times. The patient immigrated to the United States from Hong Kong several years earlier, with the last travel there 4 years previously. The patient reported having chronic malaria and symptoms for 8 years. The patient had a history of leukemia and was receiving chemotherapy. The patient was asked about travel to mainland China and other Asian countries, which he stated had not occurred. He did not have a history of blood transfusion or organ transplantation. His medical chart was reviewed, and no additional information was found. Rarely, *P. malariae* parasitemia has been documented to persist for years after initial infection (*59*–*63*). Explanations for this illness include prolonged *P. malariae* persistence or nondisclosed travel.

#### Fatal Cases

In 2017, seven fatal malaria cases were reported. All seven patients reported recent travel to Africa.

**Case 1.** A White man aged 65 years who had not used malaria chemoprophylaxis while traveling in several African countries for tourism experienced fever, chills, and headache during the flight back to the United States and went immediately to an emergency department, reporting both fever and recent travel in countries where malaria is endemic. No workup for malaria was performed, although it was noted that he had thrombocytopenia and an increased anion gap; he was sent home. He returned 2 days later with worsening thrombocytopenia, renal failure, and acidosis, and was admitted. A thick and thin blood smear for malaria diagnosis was ordered on the first day of his second evaluation; however, blood was not drawn until the following morning and results were not available until the evening. The blood smear was positive for malaria parasites with no species reported; the percentage parasitemia was reported as high but was not initially quantified. The patient was initially treated with doxycycline PO and a subtherapeutic dose of atovaquone-proguanil PO because of limited supply of the antimalarial. CDC was contacted, and intravenous artesunate arrived 3.5 hours after the call. After 2 doses of IV artesunate, repeat blood smears showed that the parasitemia decreased from 30% to <1%. However, renal failure and acidosis continued to worsen. The patient had a cardiac arrest on the evening of the third day of his hospital admission, and resuscitation attempts were unsuccessful.

**Case 2.** A White man aged 62 years traveled to multiple countries in West Africa for tourism and did not take chemoprophylaxis to prevent malaria. Approximately 8 days after returning to the United States, the patient developed weakness and confusion. On the sixth day after symptom onset, he sought care at an emergency department and was admitted to the intensive care unit (ICU) around 8 p.m. with worsening confusion and weakness, which rapidly progressed to lethargy and respiratory failure. The patient reported no fever or bleeding. He experienced septic shock and received intubation to protect his airway along with mechanical ventilation. Treatment included broad-spectrum antibiotics. At approximately 1:30 p.m. the following day, the clinical staff were notified that the patient had a *Plasmodium* species infection; no species or percent parasitemia were reported at this time. Antimalarial medication, including intravenous quinidine, was ordered and administered at 6:25 p.m. on the second day in the ICU. While hospitalized, the patient experienced hemolytic anemia (with an Hb decrease from 11 g/dL to 7.9 g/dL), acute respiratory distress syndrome, acute kidney injury, acidosis, thrombocytopenia, and multiorgan failure. His family refused blood product transfusion on his behalf because of religious reasons. During the evening of the second day of his admission, he suffered a cardiac arrest, and cardiopulmonary resuscitation was not attempted per family wishes. The patient died at 9:55 p.m. on the second day in the ICU. Four days after his death, the final laboratory report showed *P. falciparum* infection with 90% parasitemia, although this result was not externally validated by a reference laboratory.

**Case 3**. A White man aged 50 years traveled to Burkina Faso for 10 days in November 2017 on a mission trip and did not take malaria prophylaxis. A few weeks after returning, he experienced fever and fatigue but did not seek medical attention. A week after the start of his symptoms, his daughter found him at home with altered mental status and a bruise on his forehead. She took him to an emergency department, where the workup revealed acute kidney injury, jaundice, and a positive malaria smear, with no species or parasitemia reported. A CT scan of the head showed no abnormalities. The patient was administered intravenous clindamycin and early the following day was transferred to another hospital, where he was admitted to the ICU and was given IV quinidine gluconate and IV doxycycline for treatment of severe malaria. Shortly after starting quinidine, a widened QRS complex was observed on an electrocardiogram, and quinidine toxicity was suspected. IV artesunate was requested from CDC and administered. However, the patient’s condition deteriorated, requiring intubation and mechanical ventilation, vasopressor support, and continuous renal replacement therapy for acute kidney injury. The patient died on the eighth day after being hospitalized. The malaria smear at the initial hospital was sent to an outside laboratory, and results available later revealed *P. falciparum* infection with 16% parasitemia.

**Case 4.** A White man aged 76 years with a history of lymphoma, autoimmune hemolytic anemia, diabetes, and chronic kidney disease traveled to Cameroon for 1 month with unknown adherence to atovaquone-proguanil prophylaxis. He experienced fever 6 days after returning. He sought medical care on day 12 of the fever, received a diagnosis of possible uncomplicated malaria, was prescribed oral doxycycline for possible tickborne infection, and was discharged home. Malaria smears were sent to an outside laboratory, and results were available 3 days later, indicating *P. ovale* infection with 0.02% parasitemia. Although PCR was conducted, speciation was unsuccessful, and the result was documented as “*Plasmodium* present.” After receiving these results, the health care providers attempted to contact the patient with the results of the test so that he could begin treatment with appropriate antimalarials. He was found that same day unresponsive at his home and was brought to the emergency department. He was admitted to the ICU, received intubation and mechanical ventilation for acute respiratory failure, was administered vasopressors for hypotension, and experienced both renal failure and acidosis. The patient received intravenous doxycycline and quinine, administered by nasogastric tube. The patient died 1 day later. Because of the very low parasite load, cause of death was believed to be due to cardiogenic factors, unrelated to malaria. After a review of the medical records, this patient was classified as having uncomplicated malaria.

**Case 5.** A Black woman aged 56 years had traveled to Nigeria; whether she took chemoprophylaxis to prevent malaria is unknown. Approximately 3 weeks after returning to the United States, she was admitted to the hospital for fever and altered mental status, and she left against medical advice. A few days after being home, where she lived alone, she experienced bilateral lower extremity weakness and spent 4 days on the floor of her bedroom and kitchen, until a family member found her. Emergency medical services were called, and the patient was transport to a hospital (different from the one to which she had originally been admitted) by ambulance at 2:40 p.m. The patient had a history of hypertension, non–insulin-dependent diabetes mellitus, and morbid obesity. The emergency department workup showed that she had jaundice, sepsis, generalized weakness, acidosis, anemia, thrombocytopenia, and acute kidney injury. The woman was admitted and treated for severe lactic acidosis caused by sepsis and liver failure. Treatment included continuous renal replacement therapy, fluids, antibiotics, and vasopressors. At 11:18 a.m. the following day, the pathologist informed the clinical team that abnormal ring form inclusions were identified in red blood cells, compatible with *Babesia* or *Plasmodium* species. While the final diagnosis was being confirmed, the team decided to treat the patient for severe malaria, and IV quinidine was administered on her second day of treatment. Approximately 1 hour after starting the quinidine, the patient had hypotension, worsening acidosis, and an increased need for vasopressors. She also experienced obtundation and agonal breathing and received intubation and mechanical ventilation. The woman then experienced cardiac arrest, and attempts to resuscitate her were unsuccessful. She died the day after she was admitted to the hospital. Subsequent PCR testing confirmed that she was infected with *P. falciparum*.

**Case 6.** A Black man aged 44 years traveled to Nigeria for 3 weeks to visit family and friends and did not take chemoprophylaxis to prevent malaria. Two days after his return to the United States, he experienced symptoms including fever, headaches, body aches, dizziness, vomiting, and dark urine. He sought care 6 days later and received a diagnosis of severe *P. falciparum* malaria with multisystem organ failure. The patient initially was treated with IV quinidine, intubation with mechanical ventilation, and vasopressors. He received multiple blood transfusions and hemodialysis. Because of QT prolongation, quinidine was replaced with artesunate. The percent parasitemia dropped from 10% to undetectable with treatment; however, the patient remained critically ill and experienced multisystem organ failure. On hospital days 4 and 5, he experienced three cardiac arrests. Attempts at resuscitation after the third arrest were unsuccessful, and the patient died.

**Case 7.** A White man aged 78 years traveled for 2 weeks to Tanzania to visit friends and relatives. No chemoprophylaxis was taken to prevent malaria. The patient developed symptoms the day he returned to the United States. He was admitted to the hospital and received a diagnosis of *P. falciparum* malaria by blood smear and RDT, with 33% parasitemia. The patient had cerebral malaria and was treated with IV quinidine, quinine PO, doxycycline (route unknown), and atovaquone-proguanil PO. The patient died 4 days after symptom onset. Limited additional details are available for this case because the medical record was not provided for review.

## Discussion

The number of persons who received a malaria diagnosis in the United States has been increasing since the mid-1970s, with the highest number of cases since 1971 reported in 2016 (2,078) and 2017 (2,161) (Table 1). Two thirds of all malaria cases imported in 2017 were from West Africa, a trend that has been observed in other settings (*64*–*66*). *P. falciparum* infections, which cause the most severe disease and greatest risk for death, account for 70% of all imported cases in the United States. Malaria occurring among U.S. civilians accounts for the increase in cases over the last four and a half decades.

Antimalarial chemoprophylaxis medications are effective at preventing malaria, and adhering to an appropriate chemoprophylaxis regimen is the best way for travelers to prevent this potentially life-threatening illness. Among U.S. civilians, those traveling to visit friends and relatives are the most common group to acquire malaria, and they are less likely than other types of travelers to have taken any chemoprophylaxis (24.1% compared with 35.2%, among VFR and non-VFR travelers, respectively). Although pretravel health consultations are necessary to obtain prescription antimalarial medications tailored for the person’s age, pregnancy status, destination country, preferences (e.g., daily or weekly drug regimen), tolerability of potential side effects (e.g., sun sensitivity for doxycycline and neuropsychiatric disorders for mefloquine), and cost, many travelers do not seek pretravel consultations (*67*,*68*). In addition, because antimalarial medications are often not covered by insurance, out-of-pocket costs can prevent U.S. travelers from initiating chemoprophylaxis (*69*–*71*). Recently, focus groups of West African immigrant VFR travelers were convened to determine barriers to malaria prevention (*71*). Findings indicated that although VFR travelers are concerned about pretravel health care and malaria prevention, they face barriers to obtaining and using chemoprophylaxis. These barriers include unpredictable and sometimes substantial monetary costs related to the health care visits and medications, distrust of the U.S. health care system, and belief that physicians in the United States are not knowledgeable about malaria and other infectious diseases to which they might be exposed. Before travel, VFR travelers cite competing priorities for their time and finances such as undertaking prevention measures against other illnesses and budgeting for travel-related expenses, such as purchasing supplies and gifts. During their trip, VFR travelers face cultural pressures to fit in and not to inconvenience their hosts with vector control prevention measures. Traveling at the last minute for a family illness or death also can limit their ability to acquire malaria chemoprophylaxis (*71*). VFR travelers, compared with non-VFR travelers, had statistically longer durations of travel (7 weeks versus 3.8 weeks) (*68*). Insurance policies, especially Medicaid, which covers a large proportion of low-income immigrants, often have dispensing policies that limit the number of days for which a medication can be dispensed, and many insurance policies do not offer a prescription override for a vacation (i.e., allowing a prescription to be filled for more than a 30-day supply) (*70*). The focus groups that were convened indicated that VFR travelers sometimes ran out of the chemoprophylaxis antimalarial that they obtained for their trip, resulting in inadvertent nonadherence (*71*). In one study, cross-sectional surveys were conducted among U.S. resident travelers in four different categories: 1) VFR travelers in the community settings, 2) VFR travelers with recent malaria diagnoses, 3) VFR travelers attending a travel clinic, and 4) non-VFR travelers attending a travel clinic. The study objectives were to assess and compare malaria prevention knowledge, attitudes, and practices among these groups (*68*). The findings indicated that even among those who obtained chemoprophylaxis medications at a travel clinic, VFR travelers were less likely to adhere to the medication regimen (83.0%) compared with non-VFR travelers (98.7%). VFR travelers with recent malaria diagnoses were less concerned about malaria risk than other groups of VFRs and were more likely to have taken five or more previous trips to sub-Saharan Africa (*68*). This suggests that having taken previous trips that did not result in malaria decreased the perception of risk for a malaria infection overall.

Numbers of imported malaria cases have been increasing for decades, reflecting increasing rates of U.S. residents traveling internationally and non-U.S. residents who travel to the United States as visitors or immigrants. Malaria is a preventable infection, and reducing the number of imported cases is important. Prevention efforts for persons traveling from the United States should include mosquito avoidance but most importantly should include chemoprophylaxis. Accurate data are lacking regarding the proportion of travelers from the United States who are returning to the country of their or their family’s origin. However, persons traveling for the purpose of VFR made up approximately 60% of U.S. civilian patients in 2017; targeting malaria prevention efforts to this group could protect U.S. civilians from acquiring malaria. The insight from recent work to address malaria prevention among VFR travelers suggests that the motivations, situations, and health care environments for VFR travelers are complex, and improving access and adherence to malaria prevention, especially chemoprophylaxis, will require a multifaceted approach (*68*,*70*–*72*). Health care disparities for immigrant communities and limited access to travel clinics that are knowledgeable about and can provide specialized care for their unique needs are barriers to preventive pretravel care. Interventions could include community-level efforts to increase risk awareness, especially for immigrants who have been established in the United States for years and for repeat VFR travelers. Persons who are educated about the risk for malaria illness and the safety and effectiveness of malaria chemoprophylaxis can become motivated to take the medication correctly for the duration of their exposure (*73*–*75*). Resources for health care workers to discuss malaria prevention among VFR travelers is available on the CDC website (https://www.cdc.gov/malaria/travelers/vfr.html). Health insurance policies often do not provide coverage for potentially life-saving antimalarial chemoprophylaxis medications (*70*). An adult course of antimalarial chemoprophylaxis medications for a 14-day trip (including pretravel and posttravel doses) was estimated to cost between $26 for doxycycline and $48 for atovaquone-proguanil (*69*). Even the most inexpensive regimen (doxycycline) for an individual VFR traveler traveling for 7 weeks could be cost prohibitive at $91, which would be even more expensive if an entire family were to travel. These preventive costs are negligible compared with the expense of an average malaria patient’s hospitalization ($25,789) (*76*); inpatient costs for severe malaria care and treatment can approach $300,000 (*69*). Expanded health insurance coverage of preventive chemoprophylaxis medications would likely extend access to these medications to non-VFR travelers as well (*69*).

In 2018, FDA approved tafenoquine (Arakoda) for malaria chemoprophylaxis in nonpregnant persons aged ≥18 years who are not G6PD deficient as confirmed by a quantitative G6PD test (*77*). Arakoda chemoprophylaxis offers protection from acute *Plasmodium* infections, in addition to protection from relapsing infections. The recommended regimen is a loading dose taken once daily for 3 days before travel (200 mg daily for 3 days), then a weekly dose while traveling in the area where malaria is endemic (200 mg/week), followed by a single dose (200 mg) during the week after departing the malaria transmission area. The most common side effects are gastrointestinal disturbances, dizziness, and headache (*78*). Hemolytic anemia can occur if Arakoda is used by persons with G6PD deficiency, and rare psychiatric adverse reactions were observed in persons with a history of neuropsychiatric illness; hypersensitivity reactions are possible. At higher-than-recommended doses, a vortex keratopathy not affecting vision was observed but resolved within 1 year of stopping the drug (*78*). Adverse reactions to this new antimalarial should be reported through the Food and Drug Administration MedWatch site (https://www.fda.gov/safety/medwatch-fda-safety-information-and-adverse-event-reporting-program).

To prevent severe disease or death, improve prognosis, and prevent extended (and expensive) hospitalizations (*69*,*76*), persons who develop fever after travel in a country where malaria is endemic should be educated to promptly seek care for diagnosis and treatment by a medical professional. Blood smears are the mainstay for malaria diagnosis; thick blood smears are more sensitive than thin blood smears for detecting malaria parasites because a greater volume of blood is examined in each microscopic field. Thin blood smears facilitate identification and quantification of the parasite species (*79*). Blood smears should be read immediately, and the percentage of parasitized red blood cells should be calculated and provided promptly to the clinical team so that disease severity and appropriate treatment can be determined. Health care providers should assess the diagnostic and treatment resources present in their facilities, including availability at night and on weekends. The resources required to perform a malaria blood smear are similar to those for the commonly performed complete blood count with a manual differential. Qualified personnel who can perform this task should be on call 24 hours per day. Because timeliness of the diagnosis is of utmost importance, malaria tests should not be sent out to commercial laboratories, which take days or weeks to return results. In 2017, a follow-up to a 2010 nationwide survey of laboratories was conducted in the United States (*80*,*81*). Results showed that, in 2017, although most laboratories offered malaria diagnostic testing services, only 12% were in complete compliance with all the Clinical and Laboratory Standards Institute guidelines for analysis and reporting of results. Limited laboratory experience with malaria specimens can be a barrier to accurate diagnoses, and three fourths of laboratories surveyed reported diagnosing five or fewer cases annually (*81*). Despite some progress in laboratory performance, opportunities are available to further improve adherence to malaria diagnosis recommendations; specifically, 19% of laboratories did not offer malaria testing at all times, 43% did not return results within 4 hours, and 57% did not return percentage parasitemia within 6 hours (*81*). CDC provides telediagnosis assistance to laboratories and care providers who need help promptly reading the blood smears (*82*). To maintain and improve malaria and other parasitic disease diagnosis capabilities in the United States, CDC conducts training courses (https://www.cdc.gov/dpdx/training.html). The CDC Malaria Hotline provides assistance to health care providers diagnosing suspected or confirmed malaria and treating patients. To speak with the person on call for malaria consultations, call 770-488-7788 or toll-free 855-856-4713 during regular business hours, or CDC’s Emergency Operations Center at 770-488-7100 during evenings, weekends, and holidays. Additional information is available at the CDC website (https://www.cdc.gov/malaria/diagnosis_treatment/index.html).

Laboratories unable to provide immediate blood film microscopy should maintain a supply of BinaxNow malaria RDTs to assist with the initial diagnosis of malaria and develop standard procedures to ensure prompt confirmatory testing by microscopy or PCR. The limitations of the BinaxNow RDT are that the test cannot identify all *Plasmodium* species, and because the test detects parasite antigens, not viable parasites, false-positive results could occur for weeks after an infection is cleared (*41*). A microscopy blood smear should be performed for every patient assessed for malaria; optimal case management requires an assessment of the percent parasitemia to select the appropriate treatment for either uncomplicated or severe malaria, which is not possible with RDTs or PCR. Empiric treatment for malaria is not recommended because if a patient truly has malaria, then an assessment of disease severity, which includes the percent parasitemia, is not possible, which could result in improper treatment; if the patient does not have malaria, then failure to make the correct diagnosis can lead to poor outcomes (*33*).

PCR testing, which is not as timely as microscopy, is the most definitive test to determine the infecting species and should be used to confirm the results of microscopy and evaluate for mixed infections. In 2014, the Council of State and Territorial Epidemiologists released a revised malaria case definition highlighting the importance of determining the species and the percent parasitemia at the time of diagnosis and encouraging PCR testing for each case (*27*). If PCR testing is not available locally to confirm the *Plasmodium* species, then whole blood and blood smears can be sent to state public health laboratories or to CDC for diagnostic confirmation. CDC provides free diagnostic assistance to laboratories and health professionals to diagnose cases of malaria, which includes PCR testing and drug resistance marker analysis (*82*). Increasing the proportion of cases with species confirmation and drug resistance marker analysis will improve the epidemiological understanding of malaria diagnosed in the United States. Specifically, drug resistance marker surveillance in the United States might be able to detect changes in the prevalence of drug resistance markers in the countries of malaria acquisition, in which case, the U.S. treatment guidelines could be revised. In addition, understanding the regional molecular profiles of parasites acquired worldwide could provide additional evidence that would help explain malaria cases investigated in the United States, especially cryptic cases of malaria. Public health laboratories should consider developing standardized procedures for sending blood samples from persons with malaria to CDC for molecular surveillance monitoring.

Because uncomplicated malaria can rapidly progress to severe malaria, especially in nonimmune persons, persons should seek care immediately after symptoms begin and notify health care providers of their travel history, even if travel was not recent. Once malaria has been diagnosed, prompt initiation of treatment is critical. Any delay in the diagnosis and treatment of malaria can result in life-threatening complications, regardless of the effectiveness of the treatment regimen. In 2017, six of seven fatal cases had information on the number of days between illness onset and hospital admission; four of these patients sought care 6 days after illness onset, and one patient 16 days after onset. Although one patient who died sought care the same day as illness onset, the health care team discharged him without a malaria evaluation; when he returned 2 days later with worsening symptoms, the blood smear testing was delayed, and treatment for severe malaria was not started until 4 days after illness onset. This death might have been prevented if diagnosis and appropriate treatment had been promptly initiated.

Health care providers should always consider malaria in the differential diagnosis for a fever of unknown cause, especially among persons who originated from a country with endemic malaria, even if no recent travel is reported. Patients who have had blood transfusions should be evaluated for malaria if they experience a fever. After international outbreaks of infectious diseases, such as Ebola virus disease, Zika virus, and SARS-CoV-2 (coronavirus disease 2019 [COVID-19]), CDC and public health officials have investigated potential cryptic malaria cases among patients who did not disclose a complete or accurate travel history (CDC, unpublished data, 2020). For most of the potential cryptic cases, nondisclosed travel was discovered (CDC, unpublished data, 2020); sometimes a passport review or an inquiry into official travel records can be helpful to identify nondisclosed travel.

The best approach to prevent severe malaria complications is to provide prompt and appropriate treatment. The choice of an antimalarial treatment regimen should be based on several factors, including the probable geographic origin of the parasite, the *Plasmodium* species, the percent parasitemia, and the patient’s pregnancy and clinical status (*31*,*35*). Oral regimens are less effective than parenteral regimens for treatment of severe malaria and are not the standard of care. Severely ill patients should be treated aggressively with parenteral antimalarial therapy to ensure rapid adequate drug levels, with the goal to decrease parasitemia to <1% as soon as possible to minimize the likelihood of complications or death (*31*). Quinidine gluconate was the only FDA-approved medication for parenteral malaria therapy in 2017; however, as of April 2019, quinidine gluconate is no longer available in the United States (*83*). Starting in April 2019, CDC made intravenous artesunate, the WHO-recommended first-line drug for severe malaria, available through an investigational new drug (IND) protocol for every patient in the United States with severe malaria (*57*,*84*). Artesunate is stocked in several sites in the United States and can be quickly shipped free of charge to the major airport closest to the requesting hospital (*57*,*84*). To obtain artesunate for a patient with severe malaria, health care providers should call the CDC Malaria Hotline at 770-488-7788 or toll-free at 855-856-4713, Monday–Friday, 9 a.m.–5 p.m., Eastern Time. After hours, clinicians should call 770-488-7100 and ask to speak with a CDC Malaria Branch clinician. In May 2020, the FDA approved intravenous artesunate for treatment of severe malaria. Until this product is commercially available and widely distributed, CDC will continue to distribute IV artesunate via the IND protocol (*85*). Travelers and health care providers are encouraged to use CDC resources on malaria prevention and treatment (Table 8), and health care providers can contact the CDC Malaria Branch for assistance with diagnostic or case management needs. Preparedness plans for hospitals, including stocking antimalarials for treatment and malaria RDTs for after-hours testing, informing pharmacies and clinical care teams on the procedures to request artesunate from the CDC, and planning logistics to retrieve artesunate from the airport, should minimize delays to diagnosis and treatment (*86*).

**TABLE 8:** Resources for malaria chemoprophylaxis, diagnosis, and treatment recommendations

Subject	Source	Availability	Contact information
**Chemoprophylaxis**	CDC Traveler’s Health Internet site (includes online access to *Health Information* *for International Travel*)	24 hours/day	https://wwwnc.cdc.gov/travel
	*Health Information for International Travel* (*Yellow Book)*	The latest edition is available for sale from Oxford University Press (https://global.oup.com/academic) and from major online booksellers	800-451-7556 https://global.oup.com/academic https://wwwnc.cdc.gov/travel/page/yellowbook-home
	CDC Malaria Branch website with malaria and prophylaxis information, by country (Red Pages)	24 hours/day	https://www.cdc.gov/malaria/travelers/country_table/a.html
**Diagnosis**	CDC website: Malaria Diagnosis (United States)	24 hours/day	https://www.cdc.gov/malaria/diagnosis_treatment/diagnosis.html
	CDC Division of Parasitic Diseases and Malaria diagnostic Internet site (DPDx)	24 hours/day	https://www.cdc.gov/dpdx/index.html
	CDC Division of Parasitic Diseases and Malaria diagnostic CD-ROM (DPDx)	Order by e-mail from CDC Division of Parasitic Diseases and Malaria	dpdx@cdc.gov
**Treatment**	CDC website: Malaria Treatment (United States)	24 hours/day	https://www.cdc.gov/malaria/diagnosis_treatment/treatment.html
**Clinical advice**	CDC Malaria Hotline	***For clinicians and blood banks only*** 9:00 a.m.–5:00 p.m. Eastern Time, Monday–Friday After hours, on weekends and holidays	770-488-7788 or toll-free 855-856-4713 770-488-7100
**Malaria questions**	CDC INFO	***General public*** 8:00 a.m.–8:00 p.m. Eastern Time, Monday–Friday	CDC INFO 1-800-CDC-INFO (1-800-232-4636)

Patients with severe malaria were significantly more likely to have been inappropriately treated compared with those patients with uncomplicated malaria (46.2% versus 24.3%, respectively), and 25.9% of all pregnant women received treatment inconsistent with the CDC guidelines for treatment. Health care providers should be familiar with prevention, recognition, and treatment of malaria and are encouraged to consult appropriate sources for malaria prevention and treatment recommendations (Table 8).

Since 2000, the case-fatality rate from malaria has been <1.0%, with an average of 0.41%. In 2017, seven persons with malaria died, a case-fatality rate of 0.32%, which was equivalent to that in 2016 (seven deaths; case-fatality rate: 0.34%). In 2017, six of seven patients who died were infected with *P. falciparum,* and one with *P. ovale*. The patient infected with *P. ovale* did not have severe malaria and died of complications related to other underlying conditions; this patient reportedly took chemoprophylaxis with unknown adherence. None of the other fatal cases took chemoprophylaxis for malaria prevention.

The proportion of undetermined species in 2017 is the lowest reported during 2012–2017 (10.5% in 2017). At the same time, the proportion of *P. falciparum* infections (70.5%) in 2017 is the highest recorded for 2012–2017, and since 2012 on average, 104.7 additional *P. falciparum* infections occurred each subsequent year. Because *P. falciparum* infections tend to have higher parasite densities than other species and have more recognizable features, they might be easier to diagnose than other species. Travel to the African region likely contributes to the large proportion of *P. falciparum* infections; the United Nations World Tourism Organization reported an overall increase in travel rates to sub-Saharan Africa, with 41.0 million international tourist arrivals reported for 2017, an increase from 38.9 million in 2016 (*87*). Likewise, flights from the United States to Africa among U.S. citizens increased by approximately 12,300 per year during 1996–2017 (*49*). During 2016–2017, the outbound flights from the United States to Africa increased by 23,900 (*49*). An analysis of the GeoSentinel global surveillance network, which monitors travel-related illness, indicated that 83% of travelers who contracted malaria were exposed in sub-Saharan Africa (*88*). Likewise, a meta-analysis of data during 2005–2016 showed that among imported malaria infections in 40 countries where malaria is not endemic, 56% of cases were from West Africa (*66*).

Although 99.7% of cases in the United States were classified as imported, in 2017, two cases of TTM occurred in the United States; in 2016, one TTM case occurred, after a 4-year period during which no cases of TTM were documented (*11*). The United States does not have a licensed test to screen donors for malaria to protect the blood supply; therefore, FDA recommends that donor history questionnaires be used to determine eligibility for donation and defer those who do not qualify. During 2017, criteria for donor deferral were 1) a 1-year deferral for permanent residents of countries in which malaria is not endemic, after travel to a country in which malaria is endemic; 2) a 3-year deferral for persons with a history of malaria illness, after they are free of symptoms; 3) a 3-year deferral for persons who lived for 5 or more years in a country in which malaria is endemic (potential donors must have remained free from malaria and symptoms suggestive of malaria during this time); and 4) a 1-year deferral for previous residents of countries with endemic malaria, who have lived in countries without endemic malaria for at least 3 consecutive years, after they conduct short-term travel to an endemic country and subsequently did not have malaria or symptoms of malaria (*89*). The 3-year deferral recommendation for residents of countries in which malaria is endemic is based on data during 1963–1999, which showed that among 4,229 cases of malaria in non-U.S residents, seven patients (0.2%) experienced onset of malaria symptoms >3 years after they had left the country where malaria is endemic (*89*,*90*). After an additional interview, both donors were identified as potential sources in the two instances of TTM in 2017; they were immigrants from Africa and had traveled within the last 3 years. These cases of TTM could have been prevented if the FDA donor guidelines had been followed; however, perfectly implementing the donor history questionnaire is a challenge. One of the two donors did not test positive by PCR, which is not unexpected for asymptomatic persons with low parasitemia. Serologic assessments in TTM investigations, while not definitive to show presence of parasites, are useful when used to screen all donors in a TTM investigation and to identify those who have previously been infected with malaria (*10*).

In April 2020, FDA updated blood donor referral recommendations. Now residents of nonendemic countries will be deferred for 3 months after travel to a country where malaria is endemic, so long as they have no signs or symptoms of malaria during the deferral time. There is no change to the recommendation that persons who recently resided in countries where malaria is endemic should be deferred for 3 years. Former residents of countries with malaria transmission, who have lived in nonendemic countries for 3 or more years, can be deferred for 3 months after short-term travel, if they are free of malaria during this time (*91*).

Primaquine use to prevent relapse from *P. vivax* or *P. ovale* in nonpregnant patients remains low (29.6% among 331 *P. vivax* or *P. ovale* infections). In 2017, primaquine was the only antimalarial that was active against the dormant liver parasites to prevent relapses. During this time, the treatment recommendation was that all persons who receive a diagnosis of mosquito-acquired *P. vivax* or *P. ovale* and who are not G6PD deficient (confirmed by quantitative G6PD testing) should receive a 14-day course of primaquine to prevent relapses (*31*,*35*). Adherence to a 14-day regimen can be challenging. During 2018, FDA approved tafenoquine (Krintafel) for radical cure, also known as antirelapse treatment, and the medication became available in the United States during 2019 (*92*,*93*). Tafenoquine is an analog of primaquine, an 8-aminoquinoline, and targets *Plasmodium* hypnozoites in the liver. Like primaquine, tafenoquine is contraindicated among persons who are G6PD deficient or pregnant. Krintafel should be co-administered with chloroquine, in a single 300-mg dose, as the partner antimalarial for antirelapse therapy for *P. vivax* or *P. ovale* infections (*94*). This single-dose regimen might improve adherence to antirelapse malaria treatment. Hemolytic anemia can occur if Krintafel is used by persons with G6PD deficiency, and hypersensitivity reactions are possible (*92*). At tafenoquine doses higher than the recommended regimen, psychiatric adverse reactions were observed (rarely) in persons with a history of neuropsychiatric illness, and reversible vortex keratopathy not affecting vision was observed (*92*,*93*). Adverse reactions to this new antimalarial should be reported through the FDA MedWatch site (https://www.fda.gov/safety/medwatch-fda-safety-information-and-adverse-event-reporting-program) and through routine malaria case surveillance.

Treatment choices for pregnant women with uncomplicated malaria are limited to mefloquine or quinine plus clindamycin (*31*). An evaluation of the evidence for the use of atovaquone-proguanil during pregnancy was inconclusive due to the lack of data on birth outcomes (*95*). After a systematic review of existing data, in April 2018, CDC released a policy note to recommend artemether-lumefantrine for the treatment of uncomplicated malaria among women in the second and third trimesters of pregnancy. Artemether-lumefantrine can also be considered during the first trimester when other treatment options are unavailable and when the benefits outweigh the risks (*58*). This recommendation is consistent with WHO treatment guidelines and reflects current data on treatment efficacy and birth outcomes (*96*,*97*).

Since 2017, the guidelines for the treatment of malaria in the United States have been updated (*98*). First, the changes reflect the new treatment recommendations for pregnancy (*58*). In addition, with FDA approval of Krintafel (tafenoquine), antirelapse treatment using Krintafel is now an option when coadministered with chloroquine (*93*). Last, in line with WHO guidelines, artemether-lumefantrine is recommended as the first-line treatment of uncomplicated *P. falciparum* malaria. If artemether-lumefantrine is not immediately available, treatment with other effective antimalarial options on hand is acceptable to facilitate timely treatment of malaria.

The quality of malaria surveillance data can be limited if the data are incomplete or if definitions are incorrectly used. All sections of the malaria case report form should be completed because they provide information for examining malaria trends that are used to develop recommendations for malaria treatment and chemoprophylaxis. Local and state health departments, health care providers, and other health personnel should be vigilant in reporting complete information for malaria cases, especially for essential variables including species, residence, and country of acquisition. Incomplete reporting of antimalarial treatments and the infecting species could result in the patient being misclassified for receiving treatment according to CDC guidelines. CDC occasionally receives incomplete records for cases only reported electronically through NNDSS; these records contain basic demographic data but not the additional malaria case information from the NMSS case report form. CDC is collaborating with state and local health departments to update the NNDSS reporting platform through the NNDSS modernization initiative (NMI). Electronic submission of extended malaria data elements through NMI was initiated in early 2020; jurisdictions interested in electronic reporting for malaria can find information at the CDC NMI website (https://wwwn.cdc.gov/nndss/case-notification/message-mapping-guides.html). Jurisdictions that implement malaria reporting via the NMI surveillance system will no longer be required to submit paper case report forms, which should improve the timeliness and accuracy of malaria surveillance in the United States. In addition, in 2019, CDC developed an electronic case report form for jurisdictions to use during the transition period to electronic reporting through NMI. State and local health departments are encouraged to report cases using the electronic NMSS case report form until malaria-specific data can be received electronically through NMI.

Even though malaria is not endemic in the United States, malaria causes illness and deaths in this country, and recent cases are at an all-time high. Persons harboring *Plasmodium* parasites are capable of transmitting the disease via blood transfusion, and three such cases occurred during 2016–2017, the first time since 2011 (*10*,*11*,*13*). Imported cases of malaria can reintroduce *Plasmodium* parasites into receptive areas (*18*–*20*) where the disease is not endemic but potential vectors are present and environmental conditions can support the parasite lifecycle (*9*). Competent *Anopheles* mosquitoes and conditions that are conducive to malaria transmission exist in the United States (*99*). The most effective approach for U.S. residents to prevent malaria is to take chemoprophylaxis medication during travel to a country where the disease is endemic. Efforts to improve access to chemoprophylaxis for the high-risk VFR traveler could include education, improved access to pretravel health care, and insurance policy reform. Detailed recommendations for preventing malaria are available to the general public 24 hours per day online at https://wwwnc.cdc.gov/travel/yellowbook/2020/travel-related-infectious-diseases/malaria. In 2019, a new medication for chemoprophylaxis tafenoquine (Arakoda) became available in the United States to prevent malaria, which has a weekly dosing schedule for adults who are not pregnant or G6PD deficient. Prevention recommendations tailored for each country are available online (https://www.cdc.gov/malaria/travelers/country_table/a.html). CDC biannually publishes recommendations in *Health Information for International Travel* (commonly referred to as the *Yellow Book*), which is available and updated on the CDC Travelers’ Health website (https://www.cdc.gov/Features/TravelersHealth.html) (Table 8).

## Footnotes

* The term “United States” includes all states and territories.

† Countries that are considered West Africa include Benin, Burkina Faso, Cape Verde, Côte d’Ivoire, The Gambia, Ghana, Guinea, Guinea-Bissau, Liberia, Mali, Mauritania, Niger, Nigeria, Senegal, Sierra Leone, Togo, and West Africa, unspecified.
